# Vertical stratification and defensive traits of caterpillars against parasitoids in a lowland tropical forest in Cameroon

**DOI:** 10.1007/s00442-024-05542-x

**Published:** 2024-04-13

**Authors:** Sam Finnie, Philip Butterill, Vojtech Novotny, Conor Redmond, Leonardo Ré Jorge, Tomokazu Abe, Greg P. A. Lamarre, Vincent Maicher, Katerina Sam

**Affiliations:** 1 Biology Centre of the Czech Academy of Sciences, Institute of Entomology, České Budějovice, Czech Republic; 2 Faculty of Science, University of South Bohemia, České Budějovice, Czech Republic; 3 Department of Biology, Faculty of Science, Chiba University, Chiba, 263-8522 Japan; 4 Smithsonian Tropical Research Institute, Apartado, Balboa, 0843-03092 Ancon, Panama; 5 The Nature Conservancy (TNC), Libreville, Gabon

**Keywords:** Vertical forest gradient, Specialisation, Lepidoptera, Parasitoid, Diversity

## Abstract

Insect herbivores and their parasitoids play a crucial role in terrestrial trophic interactions in tropical forests. These interactions occur across the entire vertical gradient of the forest. This study compares how caterpillar communities, and their parasitism rates, vary across vertical strata and between caterpillar defensive strategies in a semi deciduous tropical forest in Nditam, Cameroon. Within a 0.1 ha plot, all trees with a diameter at breast height (DBH) ≥ 5 cm were felled and systematically searched for caterpillars. We divided the entire vertical gradient of the forest into eight, five-metre strata. All caterpillars were assigned to a stratum based on their collection height, reared, identified, and classified into one of three defensive traits: aposematic, cryptic and shelter-building. Caterpillar species richness and diversity showed a midstory peak, whereas density followed the opposite pattern, decreasing in the midstory and then increasing towards the highest strata. This trend was driven by some highly dense shelter-building caterpillars in the upper canopy. Specialisation indices indicated decreasing levels of caterpillar generality with increasing height, a midstory peak in vulnerability, and increasing connectance towards the upper canopy, although the latter was likely driven by decreasing network size. Both aposematic and shelter-building caterpillars had significantly higher parasitism rates than cryptic caterpillars. Our results highlight nuanced changes in caterpillar communities across forest strata and provide evidence that defences strategies are important indicators of parasitism rates in caterpillars and that both aposematic and shelter-building caterpillars could be considered a “*safe haven”* for parasitoids.

**Supplementary Information:**

The online version contains supplementary material available at 10.1007/s00442-024-05542-x.

## Introduction

Approximately 75% of all terrestrial trophic relationships involve insect herbivores, host plants, and parasitoids (Slinn et al. 2018) with the vast majority of these interactions occurring in tropical forests (de Souza Amorim et al. 2022). When studying these interactions, it is imperative to consider the entire vertical gradient of the forest, as many of these interactions happen high up in the canopy (Schowalter and Chao 2021). Due to the inaccessibility of the canopy, the majority of studies that focus on assemblages of insects in tropical forests are limited to saplings in the understory or focus on communities occurring solely on focal tree species or individuals. The few studies on caterpillar communities and their parasitism rates that include the entire vertical gradient of the forest, generally divide the forest into a maximum of three strata, understory, midstory and canopy (Šigut et al. 2018; Seifert et al. 2020a). Segregating the forest in this way gives us an oversimplified view of the vertical changes in insect communities and only allows direct comparisons between broadly defined strata, masking potential patterns that occur across the entire vertical gradient of the forest. Tropical forests are multi-layered ecosystems where the spatial dynamics of different tree species across vertical strata create a mosaic of microhabitats that alter the community structure of insect inhabitants at a nuanced level and they should be viewed as such (Moffett 2013). Here, we divide the forest into multiple, equally sized strata which allows us to uncover incremental patterns and changes whilst still providing an objective, standardised method of investigating the stratification of insect communities.

Lepidopteran caterpillars are the ideal study group when comparing communities of herbivorous insects across vertical gradients. This highly diverse order has an estimated 255,000 extant species, with over 150,000 already described, and is one the largest radiations of phytophagous insects (Menken et al. 2010; Mitter et al. 2017). Being apterous and having limited mobility allows caterpillars to be sampled with relative ease and ensures that they are likely to be found on their associated host plant within the vertical stratum that they occur at naturally.

In tropical forests, it is likely that the midstory, with its abundant young foliage, favourable climatic conditions and increased overlap of host plant species and microclimates (Basset 2001; Hirao et al. 2009) leads to an increase in caterpillar species richness, diversity, and density, although this has never been explicitly studied. In contrast, the harsh weather conditions (Ulyshen 2011; Nakamura et al. 2017), and reduced foliage quality (Coley and Barone 1996; Murakami et al. 2005) in the upper canopy, lead to a decline in caterpillars populations. Additionally, in tropical adult lepidopteran assemblages, neighbouring strata have the highest similarity likely due to the overlap in biotic (e.g. plant species composition and leaf quality) and abiotic (e.g. light penetration, temperature humidity and wind speed) conditions (Intachat and Holloway 2000; Schulze et al. 2001). Changes in caterpillar composition at higher taxonomic levels also shape the vertical stratification of caterpillar assemblages due to family-specific height preferences (Brehm 2007; Smedt et al. 2019). Larger caterpillars are more prevalent in the understory and smaller species are more prevalent in the canopy (Seifert et al. 2020a). This pattern is frequently observed in insects from tropical systems (Wardhaugh 2014). Specialisation in host plant caterpillar networks has been observed to increase towards the upper canopy (Seifert et al. 2020a). This is likely due to the fewer plant species and more specialised caterpillar species present in these upper strata. The trend is driven by the dominant feeding guild of shelter-building caterpillars (i.e. caterpillars that construct protective structures for themselves), which are generally more specialised than exposed feeders (caterpillars feeding openly on their host plants) (Corff and Marquis 1999; Seifert et al. 2020a). Despite these findings, there remains a scarcity of studies on the vertical stratification of larval Lepidoptera, with the majority focussing solely on adult communities (e.g. Schulze et al. 2001; Stork and Grimbacher 2006; Ashton et al. 2016; de Souza Amorim et al. 2022) or temperate forests (e.g. Šigut et al. 2018; Seifert et al. 2020a). Addressing this gap, our study aims to provide a novel insight into the subtle variations within caterpillar communities to uncover fine-scale changes across a vertical tropical forest gradient.

Previous studies of parasitism rates on caterpillars are often derived as a by-product from large-scale caterpillar rearing experiments in which parasitoids would often emerge from reared caterpillars. These experiments were primarily designed to assess herbivore communities, and to compare between herbivore feeding guilds. As a direct outcome of this focus, caterpillar hosts were classified into either exposed or concealed feeders. These studies have shed light on how these guilds can vary in their susceptibility to parasitoids and other predators, thereby shaping their distributions across vertical strata (e.g. Hrcek et al. 2013; Šigut et al. 2018). However, feeding guilds are not the sole determinants of parasitism rates in caterpillars. Defensive traits, such as aposematism, crypticity, and shelter-building also play a significant role.

Aposematic caterpillars use visual and chemical signals to advertise their unpalatability, often sequestering toxins from their host plants to deter predators (Aslam et al. 2020). However, this chemical sequestration may compromise the caterpillar’s immune response to parasitoids rendering them more susceptible to parasitism. Interestingly, it is this unique combination of a compromised immune system and enhanced predator protection that can make aposematic caterpillars ideal hosts for parasitoids, a phenomenon known as the ‘*safe haven*’ hypothesis (Dyer and Gentry 1999; Gentry and Dyer 2002; Smilanich et al. 2009).

Cryptic caterpillars employ specific coloration or mimicry to camouflage themselves from predators. Cryptic caterpillars, like aposematic caterpillars, are exposed feeders. However, they lack the chemical or visual defences that aposematic caterpillars possess, making them more vulnerable to predation, particularly from visually oriented predators such as birds (Tvardikova and Novotny 2012). This vulnerability might render them less suitable hosts for parasitoids. Parasitoids themselves are susceptible to intraguild predation (Frago 2016) and have been shown to prolong the developmental time of their hosts (Chen et al. 2017). This extended exposure increases the likelihood of the caterpillars being preyed upon, further reducing their suitability as hosts for parasitoids.

Shelter-building caterpillars construct physical shelters around themselves by rolling or tying leaves, creating a defensive barrier against predators and parasitoids. This defensive trait has been associated with increased parasitism rates compared to exposed caterpillars (Hawkins 1994; Hrcek et al. 2013; Šigut et al. 2018). Similar to aposematic caterpillars, shelter-building caterpillars may also provide a “*safe haven”* for parasitoids due to their enhanced protection from predators (Covarrubias-Camarillo et al. 2016). Furthermore, shelter-building caterpillars are more specialised on their host plants than exposed feeders (Menken et al. 2010), which has been linked to increased parasitism rates (Hrcek et al. 2013) Finally, shelter-builders are easier for parasitoids to locate than exposed feeders due to their sessile nature, in contrast, exposed feeders will often leave their feeding sites as they search for new leaves, making it more difficult for parasitoids to rely on olfactory cues to locate them.

Here we investigate various aspects of how a caterpillar community in a tropical forest in Cameroon was vertically structured as well as how their defensive traits effect parasitism rates, by testing three hypotheses:

1. We expect caterpillar species richness, diversity and density will be highest in the midstory due to higher amounts of foliage combined with favourable biotic and abiotic conditions (Basset 2001; Hirao et al. 2009), and lowest in the upper strata where there is expected to be reduced foliage quality and harsher abiotic conditions, that can only be exploited by specialist species (Coley and Barone 1996; Basset et al. 2003; Murakami et al. 2005; Ulyshen 2011).
2. We expect compositional turnover in caterpillar communities to increase between neighbouring strata towards the uppermost canopy, where changes in biotic and abiotic conditions become more drastic. We also expect to see increased dissimilarity between caterpillar communities with increased distance between the upper and lower strata, where conditions become more distinct. (Intachat and Holloway 2000; Schulze et al. 2001).
3. Network specialisation will increase towards the upper strata due to increased specialisation in the caterpillar community. We expect that this pattern will arise due to the greater abundance of specialised shelter-building caterpillars observed in previous studies (Corff and Marquis 1999; Seifert et al. 2020a).
4. Parasitism rates are expected to be highest in aposematic and shelter-building caterpillars that provide a “*safe haven*” for parasitoids as hosts and parasitism rates will decrease with increased canopy height where conditions become less favourable for both parasitoids and their hosts (Chaij et al. 2016; Vosteen et al. 2020).

## Materials and methods

### Study site

We conducted our sampling in a 0.1-ha plot of semi-deciduous tropical forest in the village of Nditam (province of Mbam et Kim), Cameroon in West Africa (5° 22′N, 11° 13′E and 709 m a.s.l.). Our forest plot was marked in a mosaic of late-secondary and primary forest and savannahs. We chose this patch as it was the least disturbed patch of forest in the nearby area destined for logging. Sampled tree height within the plot ranged from 4 to 42 m. There was a mean annual temperature of 29 °C, annual precipitation 2383 mm, and 72% mean annual humidity (measured by the local weather station). Sampling took place between the 1st of April and the 26th of June 2019, which corresponds with the “*light*” rainy season. This area of Cameroon is characterised by four seasons: a light rainy season from May to June, a short dry season from July to October, a heavy rainy season from October to November, and a long dry season from December to May.

### Sampling design

The plot was marked out, ensuring there were no forest edges, gaps or roads within a 150 m radius of the chosen area. All trees with a diameter at breast height (DBH) ≥ 5 cm were then felled one at a time. The trees were felled in a specific order to minimise disturbance to the surrounding trees, starting from the smallest and progressing to the largest. Immediately after felling, trees were thoroughly and systematically searched by 5–15 assistants for caterpillars, ensuring all individuals were collected including any that were displaced from the tree as it fell. All felled trees were identified to species, except for some species in the *Drypetes* genus, one *Ficus* and one *Chytranthus* that could only be identified to morphospecies (Table S6). This plot-based approach has proven successful in the assessment of communities of apterous arthropod herbivores (see Volf et al. 2019).

We recorded the exact height (measured from the base of the tree) where each caterpillar individual was located. Each caterpillar was photographed **(**Canon EOS 700D; 60 mm macro lens**)** and measured (total length in mm). Caterpillars were then placed individually in aerated rearing containers and given leaves from the host plant on which they were found. Rearing continued until either an adult Lepidoptera emerged, or the individual died. In some cases, parasitoids would emerge from caterpillars during rearing. Parasitoids and caterpillars were stored in 96% DNA grade ethanol and adult Lepidoptera were pinned for future identification. This method of no-choice rearing allows for successful associations between host plants, caterpillars, and parasitoids to be determined (Lill et al. 2002). Caterpillars were categorised into one of three groups: aposematic, cryptic, and shelter-building. A caterpillar was deemed aposematic if it was exposed, had bright or contrasting colours or if it had prominent hairs, spines, or bristles which although not always strikingly coloured, are still considered aposematic (Caro and Ruxton 2019). Cryptic caterpillars were, by default, any exposed caterpillars that were not considered aposematic due to their plain colouration or benign morphology. Shelter-building caterpillars were any concealed caterpillars that were found within a leaf tie, roll or a self-constructed case. All caterpillars were assigned exclusively to one of these categories, in rare cases, a caterpillar would exhibit aposematic characteristics but still be a shelter-builder, in these instances, they were always classified as shelter-building as their visual characteristics are redundant whilst they are concealed within a shelter. These three categories encompassed all caterpillars collected in this study.

For every sampled tree, the total tree height, trunk height, crown height and maximum crown width were measured. All leaves were stripped from each tree and categorised into mature or young leaves which were then placed into separate bags. A subset of leaves was taken randomly from each bag and were then spread over a white leaf frame (50 × 50 cm board), photographed and then weighed. The specific leaf area (SLA) for young and mature leaves was then calculated by dividing the total surface area (calculated using the software ImageJ v1.48; Rasband 2011) of the leaves on the leaf frame(s) by their total dry mass. For trees with larger leaves, multiple leaf frames were used, and their dry mass was combined to calculate SLA. The bags containing all the leaves were then weighed and total surface areas were calculated by multiplying the SLA by the total try mass. The total surface area per tree crown was calculated by combining the total surface area for all the young leaves and all the mature leaves. In some cases on very large trees, an estimated 25% or 50% of total leaves were weighed and the total was quadrupled or doubled respectively, to approximate the total weight of all leaves for that tree. Similar methods are often used to calculate total leaf area (e.g. Sam et al. 2020; Houska Tahadlova et al. 2023).

### Vertical stratification

The vertical gradient of the forest plot was divided into 8 equally sized strata of 5 m: 0–5 m, 5–10 m, 10–15 m, 15–20 m, 20–25 m, 25–30 m, 30–35 m and 35–40 m. The vertical strata of the forest are grouped into three categories: the lower strata (0-10 m), the midstory strata (10-30 m), and the upper strata (30–40 m). Within the midstory strata, we further identify the low midstory (10–20 m), the central midstory (15–25 m), and the upper midstory (20–30 m) for more specific observations. This terminology enables us to articulate general patterns across the forest’s vertical gradient without the need to reference individual strata. There were three trees that marginally exceeded 40 m in height, but this additional stratum was not included in our analyses as it contained no caterpillars, and the total surface area was deemed too small. To date there is no unified method of segmenting vertical forest layers, however, previous studies adhere to keeping each layer the same size (Parker and Brown 2000; Seifert et al. 2020a; Amorim et al. 2022). Each caterpillar was assigned to a given stratum dependent on the height at which it was found on the tree. Most tree crowns in our plot were spread across multiple strata so total leaf area of the crown was divided proportionally for each stratum. This division enabled separate estimation of caterpillar densities for each stratum. To facilitate this, the volume of a spheroid was used to approximate crown volume (*V*_*crown*_) using the equation:

$${V}_{crown}=\frac{4}{3}\pi {a}^{2}c,$$

Here, *a* is the horizontal radius of the crown (0.5 × maximum crown width) and *c* is the vertical radius (0.5 × crown height). When one of the upper or lower caps of the crown occurred within a stratum the total volume was calculated using the equation:

$${V}_{cap}=\frac{\pi {a}^{2}}{3{c}^{2}}{h}^{2}\left(3c-h\right),$$

Here, *h* is the height of the crown cap, and *a* and *c* represent the same as for crown volumes. When a stratum occurred within the centre of the crown (the top and bottom parts of the crown were not present within the stratum) then the volume was calculated by subtracting the volume of the cap above and below and subtracting their combined total volume from the total volume of the spheroid.

Additionally, we calculated the surface area of each tree trunk within a given stratum assuming each tree trunk to be cylindrical using the equation:

$${A}_{trunk}=2\mathrm{\pi rh},$$

This equation calculates the lateral surface area of the trunk where r is the radius of the trunk (0.5 × tree diameter) and *h* is the length of trunk within a given stratum. For this study, the trunk and crown were considered two distinct parts of the tree. The total height of the trunk ended where the bottom of the tree crown began. In cases where a stratum contained sections of both crown and tree trunk, leaf and trunk area were combined. This provided us with a standardised method of calculating the total amount of occupiable area, hereafter referred to as total surface area, for a caterpillar within a given stratum for each tree individual in our plot. The use of spheroids is commonplace when analysing the structure of forests (e.g. Chen et al. 2005; Walcroft et al. 2005; Seifert et al. 2020a). However, the addition of trunk surface area is a novel concept. This approach provides a comprehensive representation of the entire tree, acknowledging that branches and foliage can occur along the trunk, even before the ‘defined’ tree crown. In our study, 13% of caterpillars were found below the crown, emphasising the importance of including these areas for an accurate reflection of ecological reality and caterpillar distribution. Additionally, this method accounts for species- and age-specific trunk-to-crown ratios, ensuring a realistic representation of tree structure within our study.

### Insect identification

All Lepidoptera and parasitoid specimens were identified as far as possible taxonomically and assigned to a morphotype where appropriate based on their physical characteristics. At least one specimen from each morphotype was then barcoded at the Canadian Centre for DNA Barcoding (CCDB; Guelph, Canada) using standard Sanger sequencing protocols (Wilson 2012). In instances where a definitive identification was not possible, the ‘Barcode Index Number System’ (BIN system; Ratnasingham and Hebert 2013) was used. This method allowed us to distinguish between putative species and has been adopted in many ecological studies on Lepidoptera in recent years (e.g. Delabye et al. 2019; Hausmann et al. 2020). All preserved Lepidoptera and parasitoid specimens are deposited at the Institute of Entomology in České Budějovice (Czech Republic).

## Statistical analyses

All statistical analyses were performed using the statistical software R version 4.2.2 (R Development Core Team 2022).

Based on sample sizes, we opted not to include parasitoids in any density-related analyses or network analyses. The low number of parasitoid individuals after dividing the data into eight strata was insufficient for any robust analyses. Parasitoids were only included in analyses that specifically focussed on the percentage of individuals within a given population that were successfully parasitised, hereafter referred to as parasitism rate.

All linear models used in our analyses were developed using the ‘lme4’ package in R (Bates et al. 2014). For each model, tree individual (*N* = 142) nested within tree species (*N* = 44) were included as random factor. Both variables used as a random factor have been shown to alter caterpillar-parasitoid communities (e.g. Šigut et al. 2018). All best-fitting linear models in our analyses were tested against the null model using both Akaike information criterion (AIC) using the ‘bbmle’ package (Bolker 2017) and Analysis of Variance (ANOVA) using the ‘lmerTest’ package (Kuznetsova et al. 2017) to estimate the P value. When comparing the effects of the different caterpillar defensive traits, we did pairwise comparisons using estimated marginal means (EMMs) using the 'emmeans' package (Lenth 2023) to calculate P values between each group.

### Species richness, diversity, and community composition

Caterpillar species richness (SR) was calculated as the total number of caterpillar species per stratum. To compare diversity between strata we calculated Shannon diversity indices (H') for each strata using the ‘diversity’ function in the ‘vegan’ package (Oksanen 2010). We generated 1000 bootstrap replicates of each diversity index using the ‘boot’ package for each stratum, we then calculated the standard error of these replicates to estimate the standard error for each index.

To compare proportional composition of the most common caterpillar families (min. total abundance ≥ 100) amongst strata we used Chi squared contingency tests. For these, we adjusted the P values using the Bonferroni correction to account for multiple comparisons and reduce the risk of type I error. To compare overlap, we calculated pairwise Morisita-Horn (D_MH_) dissimilarity index (Morisita 1959; Horn 1966) values between strata of caterpillar assemblages using the ‘vegdist’ function in the ‘vegan’ package. This index is based on the abundance of species and was chosen because of its robustness to variations in sample sizes and diversities as it is less affected by the presence of rare species (Beck et al. 2013).

After the removal of singletons, to increase the robustness of richness estimates (Lim et al. 2012), individual-based rarefaction and extrapolation curves for species richness, were calculated for each stratum and between defensive traits using the ‘iNEXT’ package (Hsieh et al. 2016). Species richness estimates (SChao) were calculated for each strata based on asymptotic diversity (Chao and Jost 2015). Additionally, confidence intervals (CI) were calculated and plotted; nonoverlapping CI indicate significant differences between strata (Colwell et al. 2004).

### Caterpillar density

To ensure all comparisons between strata were standardised, caterpillar densities (individuals per m^2^ of total leaf + trunk area) were calculated for a given tree species in each stratum. Caterpillars that were found within a stratum containing a total surface area of less than 1 m^2^ of foliage for a given tree species were excluded from the dataset (3.4%). To meet the assumption of normality, we log10-transformed the density values prior to further analyses. When comparing density patterns across the entire vertical gradient, median height values of each stratum were substituted so that height could be treated as a continuous variable within our models.

Two linear mixed models (LMMs) were developed to test the density distribution of all caterpillars across strata and caterpillar defensive traits across strata. For both LMMs, a second-degree polynomial distribution was used to approximate the expected density pattern across the vertical gradient.

### Network specialisation

Quantitative, density-based interaction matrices were created for each stratum and analysed using the R package “*bipartite”* (Dormann et al. 2009). Density values were preferred over raw abundances when comparing networks to account for differences in vegetation between strata. To compare specialisation, we used three quantitative network indices that account for interaction frequencies. These indices are less affected by differences in sample size and sampling effort than qualitative indices and thus reflect the network structure more realistically (Banašek-Richter et al. 2004; Blüthgen et al. 2006). They are derived using Shannon diversity indices. We calculated weighted connectance, weighted generality, and weighted vulnerability using the ‘networklevel’ function implemented in the R package ‘bipartite’ to characterise the interactions networks for each stratum. Weighted connectance is the proportion of realised interactions measured as the proportion of links weighted by interaction frequency. Weighted generality and vulnerability are two indices that describe the feeding relationships between caterpillar species and host plants. Weighted generality indicates the average number of host plants that a caterpillar species feeds on, whilst vulnerability indicates the average number of caterpillar species that feed on a plant species. They are both weighted by interaction strength. Generality and vulnerability indicate the specialisation of a certain trophic level (resource level: vulnerability; consumer level: generality). Only plant species interacting with at least one caterpillar species were considered for all calculated network metrics.

To interpret index values for connectance, weighted generality, and weighted vulnerability, we used null model simulations of the interaction networks for each stratum. To generate null models, we used the ‘vaznull’ function available in the ‘bipartite’ package to randomise the interaction network matrix 999 times within each stratum. These null models were constrained by connectance, with marginal totals proportional to the observed ones (Vázquez et al. 2007), and we measured all network metrics in these random networks, creating a null distribution for each index. The use of null models allows us to gain a better understanding of network properties beyond what we can observe from the index values alone (Dormann et al. 2009).

To account for network size, we calculated standardised effect sizes (Z-scores) and corresponding P values for each specialisation index. This allowed us to compare the interaction networks and determine the degree of specialisation in each. An increase in Z-scores indicates an increase in specialisation between the networks, whilst a decrease in Z-scores indicates a decrease in specialisation.

### Parasitism rates

Parasitism rates were calculated for each stratum for all caterpillars and separately for caterpillars from each defensive strategy. The effect of caterpillar defensive traits on parasitism was tested by a Generalised Linear Model (GLM). An additional GLM was designed to determine whether parasitism was affected by vertical strata (i.e. median strata height).

## Results

We sampled caterpillars on a total of 142 trees (DBH ≥ 5 cm) from 44 species and 19 families growing within the 0.1 ha plot, only 1 tree species represented by a single individual could not be identified. The trees provided more than 5600 m^2^ of surface area on which the caterpillars were collected (850 m^2^ of trunk area and 4750 m^2^ of leaf area). In total, we sampled 1675 caterpillars from 248 species and 17 families. In total, 1554 caterpillars (92.8%) were successfully assigned to a stratum, and a host plant species. This included 379 aposematic caterpillars from 66 species, 543 cryptic caterpillars from 109 species, and 632 shelter-building caterpillars from 72 species (Table S1). In total, 121 individuals (7.2%) could not be categorised into a stratum and were therefore excluded from density-related analyses. Caterpillar abundance varied across strata increasing from 87 individuals in the lowest stratum (0–5 m) to 331 individuals at 10–15 m. Total surface area varied from 1241.11 m^2^ at 5–10 m to 271.20 m^2^ in the highest stratum (35–40 m) (Table 1). Amongst tree species, *Hylodendron gabunense* harboured the highest diversity of caterpillars (69 spp.) and there were 11 tree species on which no caterpillars occurred. The most abundant caterpillar species was a Crambid, likely from the genus *Coachena* (BOLD:AEE1691) with 189 individuals.

**Table 1:** Total surface area (trunk + leaf area on which caterpillars were collected), and the abundance and diversity of caterpillars and plants within each vertical stratum and for all strata combined (Total)

Stratum	Total surface area (m^2^)	Abundance	Caterpillar			Plant		
			Species richness	Family richness	Shannon diversity	Abundance	Species richness	Family richness
0–5 m	416.73	87	37	11	2.37	131	44	17
5–10 m	1241.11	243	82	20	3.38	130	44	17
10–15 m	903.70	331	87	16	3.23	80	36	14
15–20 m	913.97	304	92	18	3.31	46	23	13
20–25 m	789.08	230	73	16	3.94	33	18	10
25–30 m	457.56	172	64	16	2.83	18	11	5
30–35 m	609.85	108	44	11	2.52	10	10	5
35–40 m	271.20	67	25	7	2.30	6	6	3
Total	5603.20	1554	248	17	5.12	142	55	19

### Species richness, diversity and community composition

The most abundant families (> 100 caterpillar individuals) across the entire vertical gradient were Erebidae (405 indiv.), Geometridae (222 indiv.), Crambidae (214 indiv.), Tortricidae (137 indiv.) and Pyralidae (120 indiv.). Over 73% of all caterpillars belonged to one of these five families. Each of these families were present in every stratum, with the exception of the highest stratum (35–40 m) from which there were no caterpillars from Erebidae and Crambidae (Fig. S1). The most speciose families were Erebidae (54 spp.) and Geometridae (47 spp.) which collectively accounted for 40% of all species in this study.

**Fig. 1:**
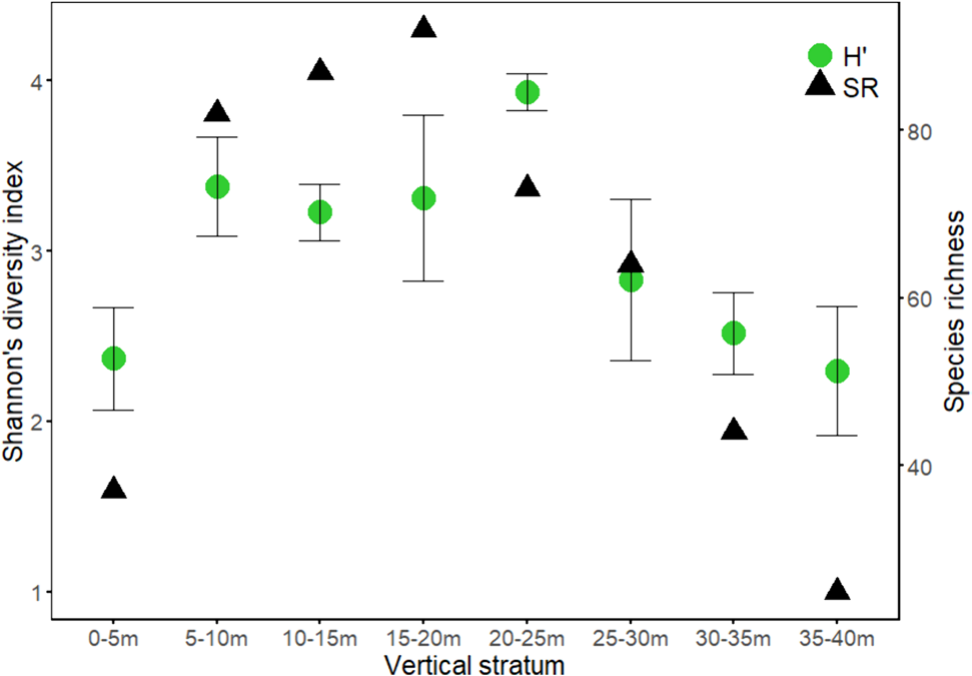
Shannon diversity index (H′) and its standard error (green dot and whiskers, left Y axis) and observed species richness (SR) (black triangles, right Y axis) of caterpillars along vertical forest strata in a tropical forest in Cameroon

Overall caterpillar species richness increased from 37 species in the lowest stratum (0–5 m) to 92 species in the at 15–20 m, where it peaked, and then declined towards the highest stratum (35–40 m, 35 species). Shannon diversity indices (H') indicate that diversity was highest (H′ = 3.94) at 20–25 m where there was also the lowest SE, and lowest (H′ = 2.30) in the highest stratum (35–40 m) (Fig. 1).

**Fig. 2:**
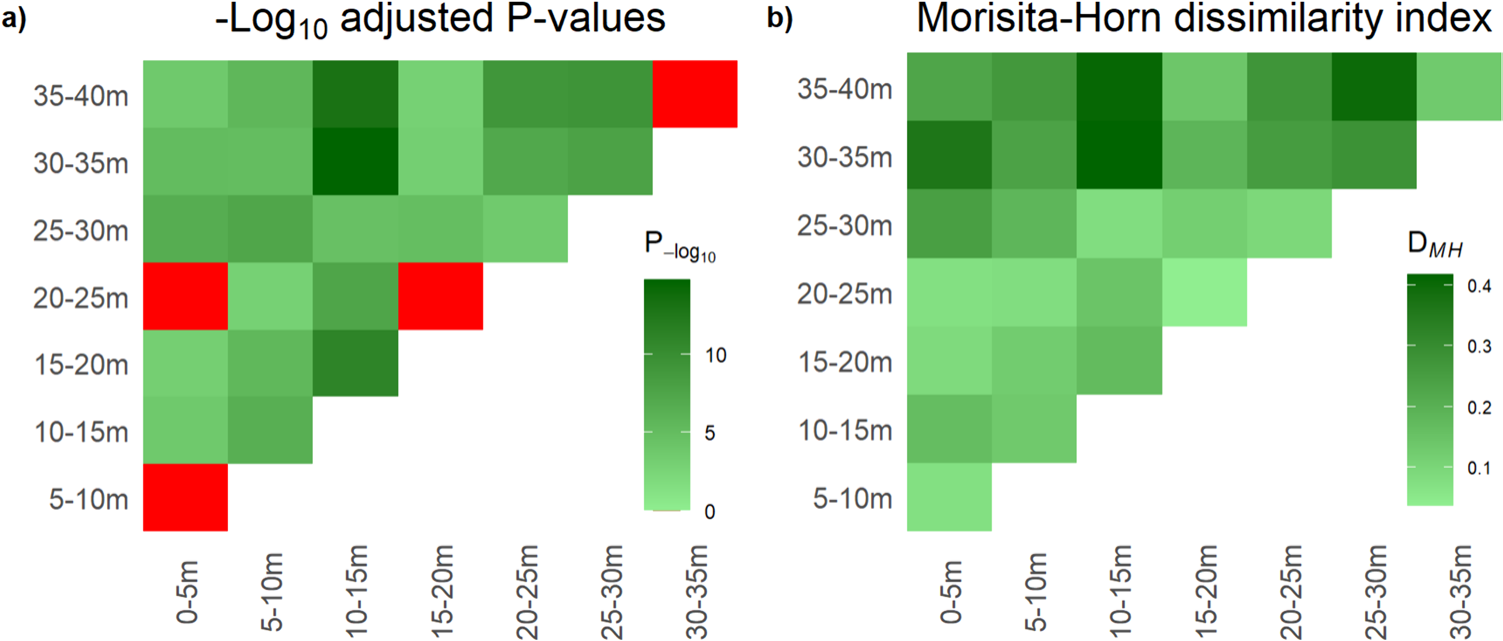
Heatmaps showing pairwise comparisons of caterpillar communities between strata across a vertical forest gradient in a tropical forest in Cameroon. Values in **a** show -log_10_ adjusted P-values from pairwise, Bonferroni corrected Chi Squared contingency tests, where darker shades indicate larger significant differences in caterpillar family proportions between strata and red boxes indicate no significant difference and **b** Morisita-Horn dissimilarity indices (D_MH_) where darker shades indicate a higher compositional turnover of caterpillar species between strata and lighter shades indicate higher overlap between caterpillar communities

Proportional abundance of the five most common families was significantly different amongst strata across the entire gradient (X^2^ = 257.9, df = 28, *P* < 0.001). Only four of the pairwise comparisons did not significantly differ, all of which were between neighbouring strata. Compositional turnover was highest between 10–15 m and 30–35 m. There was no visible trend in turnover between neighbouring strata, with increasing height (Fig. 2a). Based on Morisita-Horn dissimilarity indices, the highest compositional turnover was between 10–15 m and 30–35 m (DMH = 0.417). There was no visible trend in dissimilarity with increasing distance between strata (Fig. 2b).

**Fig. 3:**
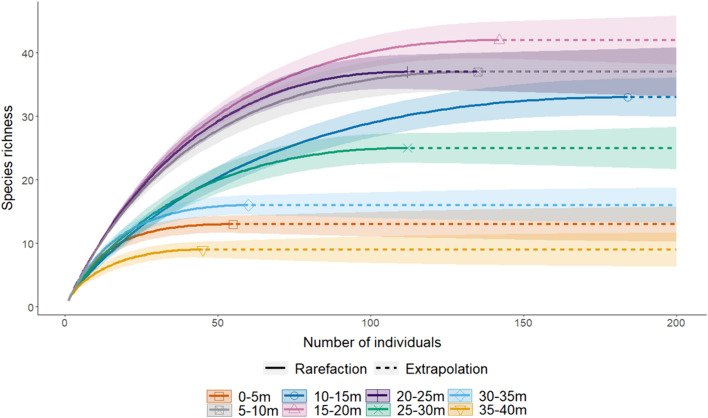
Individual-based rarefaction curves (solid lines) and short-range extrapolation (dashed lines) for the species richness of each stratum after singletons were removed. Shaded areas represent ± 95% confidence intervals, non-overlapping confidence intervals indicate significant difference

The species richness curves reveal two distinct strata groups, with an intermediate stratum between them. All strata between 5–10 m and 20–25 m are the most species-rich, with significantly more species than the other four strata. The least species-rich are the lowest (0–5 m) and two highest strata (30–35 m and 35–40 m). The third highest stratum (25–30 m) serves as an intermediate, with its species richness significantly lower than the most species-rich group and higher than the least. The asymptotic nature of all curves suggests comprehensive species sampling in each stratum (Fig. 3).

### Caterpillar density

**Fig. 4:**
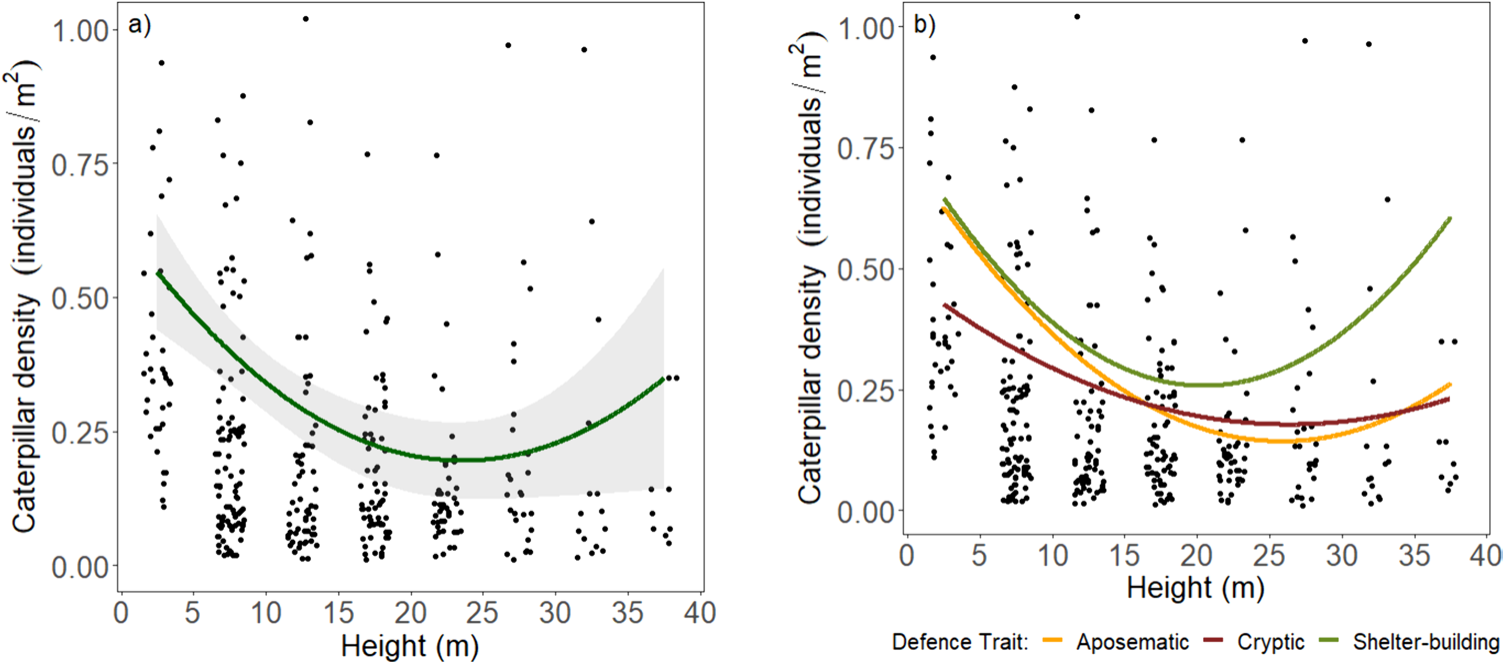
Caterpillar density across a vertical gradient in a tropical forest in Cameroon. Graph **a** shows overall caterpillar density (individual per m^2^) fitted by 2nd degree polynomial distribution ± s.e. and **b** shows caterpillar density partitioned into three defensive traits. Individual points represent the density of caterpillars for a particular tree species within a stratum. Median height values are used to approximate the height range for each stratum. In graph a) the grey area represents the standard error across the entire vertical gradient. The y-axis limits were set to 1 for ease of visualisation which led to the visual exclusion of 26 points, but all data points, including those above the limit, were included in the analysis

Overall caterpillar density had a significant 2nd degree polynomial distribution (df = 262.38, *t* = 4.67 *P* < 0.0001) where density decreased towards the central midstory and then increased towards the top of the canopy (Fig. 4a). Caterpillar density distributions between defensive traits showed no significant difference between aposematic and cryptic (*P =* 0.24), concealed and cryptic (*P =* 0.66) and aposematic and concealed (*P =* 0.06) (Fig. 4b).

### Network specialisation

**Fig. 5:**
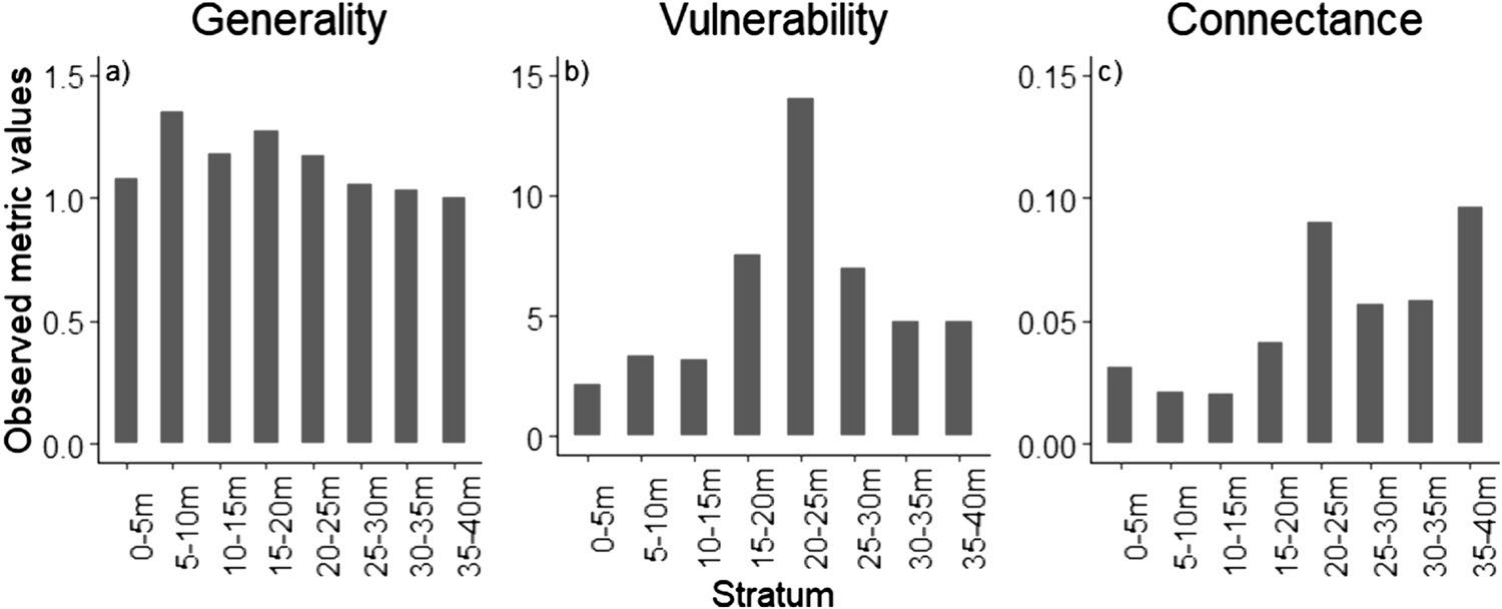
Observed metric values for generality (number of host plants per caterpillar), vulnerability (specialisation in interactions), and connectance (proportion of realised interactions) for each vertical forest stratum

Observed weighted specialisation network values showed a general, gradual decrease in generality with increasing height after an initial increase between the two lowest strata (0–5 m and 5–10 m) (Fig. 5a) Vulnerability showed a strong, midstory peak at 20–25 m (Fig. 5b). Connectance showed a broad tendency to increase with increasing height, although there is also a secondary, midstory peak at 20–25 m (Fig. 5c). Z-scores for all indices indicate increased specialisation towards the midstory and decreased specialisation towards the upper canopy (Table S2).

### Parasitism rate

**Fig. 6:**
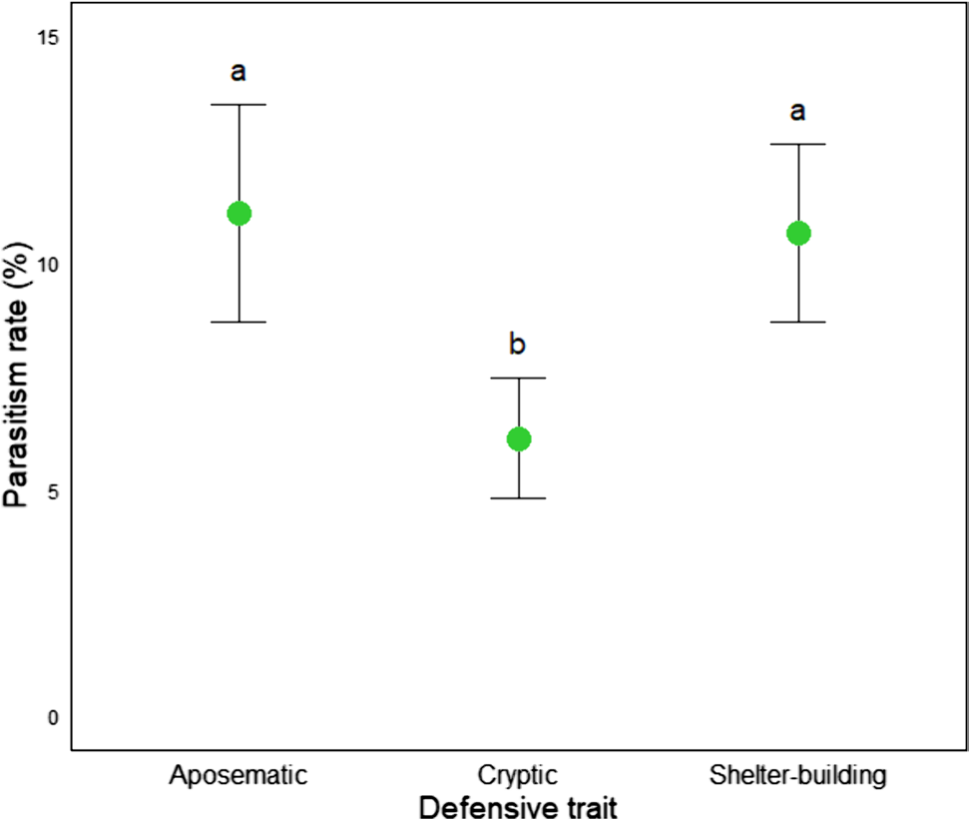
Mean parasitism rates (in %, ± SE) of caterpillars grouped by their defensive traits. The dots represent the mean parasitism rate for each group: aposematic, cryptic, and shelter-building. Whiskers represent the standard error from the mean. Different letters above the whiskers indicate significant differences between the defensive traits

The mean parasitism rate across all caterpillars was 9.5%. Mean parasitism rates were significantly higher in aposematic (11.1%, SE = 0.024) and shelter-building (10.7%, SE = 0.019) caterpillar than in cryptic (6.1%, SE = 0.013) caterpillars (odds ratio = 1.91, *P* < 0.05 and odds ratio = 1.83, *P* < 0.05, respectively). There was no significant difference between aposematic and shelter-building caterpillar (odds ratio = 1.05, *P* > 0.05) (Fig. 6). Parasitism rates showed no significant pattern across strata (*P* > 0.05) for all caterpillars (*P* > 0.05) and between defensive traits (*P* > 0.05) although parasitism was generally higher in the lower and midstory strata and lower in the upper strata (Table 2).

**Table 2:** Parasitism rates (%) defined as the percentage of parasitised caterpillars across strata for all caterpillars and between caterpillar defensive traits

Stratum	Parasitism rate (%)			
	All caterpillars	Defensive trait		
		Aposematic	Cryptic	Shelter-building
0–5 m	7.3	12.0	3.7	8.8
5–10 m	14.5	8.5	15.2	16.1
10–15 m	8.8	10.6	6.1	9.6
15–20 m	6.5	7.8	7.0	5.3
20–25 m	15.2	17.2	10.0	19.7
25–30 m	7.7	15.2	3.1	8.2
30–35 m	5.5	4.3	5.6	6.0
35–40 m	2.9	0	5.5	3.2

## Discussion

### Species richness, diversity, and community composition

By dividing the forest into multiple strata, we observed nuanced, incremental changes in caterpillar species richness and diversity across a vertical gradient. To our knowledge, this is the first study comparing the species richness and diversity of larval lepidoptera across a vertical gradient in the tropics, with previous studies focussing exclusively on adults (e.g. Schulze et al. 2001; Souza Amorim et al. 2022). We found a clear increase in both the species richness and diversity of caterpillars towards the central midstory strata and then a distinct decrease towards the upper strata. This pattern is consistent with our hypothesis (H1); however, it was not entirely driven by the ecological parameters we expected. Our initial hypothesis was that increasing foliage availability, favourable climatic conditions, and a higher overlap of host plant species would drive this pattern. However, this was not completely reflected in our results. In our study, the increase in caterpillar species appears to be mainly driven by their abundance, as the strata containing the highest number of caterpillar individuals generally have more species. Curiously, caterpillar abundance and richness were not entirely driven by available foliage, as the stratum containing the most foliage (5–10 m) had fewer species and individuals than the two midstory strata directly above. This suggests that the environmental conditions in the midstory are more favourable to caterpillars, increasing their overall abundance and diversity. Our results partially align with other studies on the vertical stratification of caterpillars from temperate forests, which found higher midstory diversity compared to the canopy, but similar or greater diversity in the understory (Hirao et al. 2009; Seifert et al. 2020a). The variation between the biotic and abiotic factors in tropical and temperate forests across vertical strata may explain this distinction. In tropical forests, the midstory has significantly higher plant diversity than the emergent canopy and higher structural complexity than the understory, thus providing more resources and niches for caterpillars (Basset 2001). In contrast to the dense canopies of tropical forests, temperate canopies exhibit a more open structure, permitting greater light penetration to the understory. This results in a less favourable environment for certain host plant species that serve as critical resources for many caterpillar species and decreasing caterpillar diversity in the understory. It is also likely that many exposed caterpillar species avoid the upper, emergent canopy where they are more susceptible to desiccation. Within the midstory, the more stable, intermediate levels of light, temperature, and humidity may be more favourable for the developmental success of caterpillars which facilitates the high diversity of caterpillars within the midstory strata in our study. It is also important to note that these results could be, in part, driven by our experimental design. Smaller saplings (≤ 5 cm DBH) were not sampled within our plot, so it is likely that some of the caterpillar community and therefore diversity in the lower strata were not fully accounted for.

Surprisingly, there were no visible trends in the increase of species turnover across the vertical gradient of our forest (H2) although there was a significant difference in the proportional abundance of caterpillar species. We hypothesised that there would be a greater turnover of species across the midstory strata compared to other neighbouring strata, driven by the larger diversity of foliage, structural complexity and distinct microhabitats conducive to different caterpillar species. Additionally, we thought that the intermediate conditions of the midstory would encourage spillover from both understory and upper canopy species, which would drive an increase in diversity. Instead, species turnover was consistently high across all strata. Large amounts of species turnover are often a result of high diversity (Coelho et al. 2018), which is certainly apparent in our study. Seifert et al. (2020a) also found that species turnover changed significantly amongst strata in temperate forest. It may be that although the strata in our study are hosts to distinct communities of caterpillars, the high diversity in all strata are overshadowing any underlying trends in species turnover across the vertical gradient in our plot. Furthermore, we expected to find increased dissimilarity between caterpillar communities with increasing distance between strata. Although there was no clearly identifiable trend, there was a tendency for comparisons between the upper strata to have higher dissimilarity values in comparison to the strata below. This is probably due to the fact that fewer species were found in these upper strata and the species that did occur in these strata were highly specialised and therefore not found in the lower strata.

### Caterpillar density

Our results reveal an intriguing, complex distribution of caterpillar density across strata, revealing novel patterns and emphasising how certain defensive traits may play an important role in shaping them. Studies comparing caterpillar density across vertical gradients in both tropical and temperate forests typically find changes across vertical strata (e.g. Basset 2001; Pontes Ribeiro and Basset 2007). The nature of these changes, however, are inconsistent between studies (Ulyshen 2011). Here we provide a novel approach by uncovering patterns in caterpillar density across multiple strata spanning an entire vertical gradient instead of comparing density in the understory, midstory and canopy (e.g. Šigut et al. 2018; Seifert et al. 2020a). Based on the literature, we expected to find the lowest caterpillar density at the top of the canopy. This is due to several factors: increased visibility and susceptibility to predation (Posa et al. 2007), higher risk of desiccation (Greeney et al. 2012), exposure to harsh weather (Basset et al. 2003), and lower leaf quality as canopy leaves are typically smaller, tougher, and have higher phenolic contents, making them less appealing to caterpillars (Coley and Barone 1996). Curiously, we found that caterpillar density actually increased towards the upper strata in our forest plot. This follows the opposite pattern to caterpillar abundance which increased towards the midstory and decreased towards the upper strata. It is possible that whilst the high availability of edible foliage in the midstory increases the overall abundance of caterpillars, it also decreases the competition between individuals feeding on the same plant, allowing them to co-exist in high abundances whilst also being at relatively low densities within this layer of the forest. In our study in particular, the pattern in density appears to be driven by a small number of shelter-building caterpillars occurring at high densities in the uppermost strata. Both Corff and Marquis (1999) and Seifert et al. (2020a) found that shelter-building caterpillars occur at higher densities than exposed feeders in the canopy. The natural history of shelter-building caterpillars makes them less susceptible to the aforementioned conditions at the top of the canopy and it is possible that certain species of shelter-building caterpillars have evolved to exploit the generally less favourable conditions in the emergent canopy and thrive where there is significantly less competition from other insect herbivores and a reduced risk from predators and parasitoids, allowing them to occur at a higher density. Predation is also an important determinant of insect distributions and has been shown to reduce caterpillar density by over 60% in a temperate forest (Singer et al. 2017) and caterpillars may preferentially locate themselves in enemy-free space (Šigut et al. 2018). Interian-Aguiñaga et al. (2022) found higher predation rates on midstory model caterpillars compared to the lower canopy and found the lowest abundance of insectivorous birds in the upper canopy. Predation rates have also been shown to increase with increasing plant diversity in tropical and temperate forests at small spatial scales (Leles et al. 2017) and the midstory contains the largest diversity of foliage. It is therefore possible that increased predation rates in the midstory strata are driving the density patterns observed in our study.

It is also important to mention that caterpillars within the upper strata are more likely to occur within a ‘*spheroid cap’* (see “Methods” section), where the crown model we used assigns less volume and therefore less leaf area. It is therefore possible that the observed increase in density towards the upper strata may be a product of our analytical design. Indeed, the use of spheroids relies on certain assumptions about the geometry of tree crowns that may not always hold true in nature. However, it is common for the volume of tree crowns to decrease towards the top of the crown where the branches and foliage of trees become smaller and less dense. Additionally, these ‘*spheroid caps’* were present across all strata as tree height varied within our plot making it unlikely that density patterns were driven by this aspect of our methodology. Equally, the addition of trunk surface areas, which were generally lower than total leaf area within a stratum, may have affected the higher densities in the lower strata. Although, as trunk heights varied to slightly below thirty metres in our study and caterpillars were found below the crown across all strata below this height, these effects should be inconsequential.

### Network specialisation

To our knowledge, this is the first study addressing specialisation patterns in host plant-caterpillar networks along a vertical forest gradient in the tropics. Our results concur with both our hypothesis (H3) and Seifert et al. (2020a) that used similar metrics in a temperate North American forest, and found that generality was highest in the understory, vulnerability was highest in the midstory and connectance was highest in the canopy, although concluded that the latter result was likely a product of small sample size. The gradual decrease of generality towards the higher strata indicates that caterpillars occupying the higher strata are more specialised. Proportionally, shelter-building caterpillars were the most abundant caterpillars in the upper canopy strata (Fig. S2), which is consistent with other studies (Corff and Marquis 1999; Seifert et al. 2020a). Furthermore, shelter-builders were also the most specialised of our three caterpillar groups with 58% of species being specialists (only being found on a single host plant species) within our forest plot (Table S3). These findings align with previous studies (Seifert et al. 2020b; Molleman et al. 2022). It is therefore likely that the natural distribution of shelter-building caterpillars has, in part, driven the overall trend in generality across the vertical gradient of the forest. However, it is worth noting that whilst there is a significant presence of shelter-building caterpillars in the lower strata, the high diversity of other generalist caterpillar species in these areas likely mitigates their impact on overall generality.

The strong, midstory peak in vulnerability, indicates that a significant proportion of caterpillar species are confined to a few host plants within this forest layer. This is exemplified in our study, where the majority of caterpillars were found on the two plant species that exhibited the highest caterpillar abundance and diversity within our plot: *Celtis philippensis* and *Hylodendron gabunense*. Together, these two species were host to 65% of all caterpillar species and 80% of individual caterpillars within the stratum with the highest vulnerability (20–25 m) (Table S5b). These species also explain the anomalous increase in connectivity within this stratum as they are responsible for so many interactions within the network. The general trend in connectedness is likely due to decreasing network size rather than increased redundance and stability in the uppermost strata as smaller networks tend to have higher connectance due to sampling effects (Pellissier et al. 2018) which is also consistent with Seifert et al. (2020a).

### Parasitism rates

In line with our hypothesis (H4), the parasitism rates of aposematic caterpillars are higher than in cryptic caterpillars and comparable to the parasitism rates of the shelter-building caterpillars within our forest plot. Aposematic caterpillars often sequester toxins from their host plants, which along with their warning colouration and morphology enhances their ability to deter predators. Parasitoids can be much more tolerant to the defensive compounds of aposematic caterpillars than generalist predators (Lampert et al. 2010). This is because the chemical sequestration of aposematic caterpillars can increase the probability of experiencing an impaired immune response, making them more susceptible to parasitoids and a safe haven for oviposition and the subsequent development of their larvae. The “*safe haven*” hypothesis (Dyer and Gentry 1999; Gentry and Dyer 2002; Smilanich et al. 2009) refers exclusively to chemically defended caterpillars. However, we argue that shelter-building caterpillars also fit into this category. Their shelters not only protect them from predators and hinder their ability to escape parasitoid oviposition, but they also create favourable environmental conditions by reducing water loss, blocking direct sunlight and wind, and reducing the chance of desiccation (Abarca and Boege 2011; Greeney et al. 2012). As hosts, shelter-building caterpillars provide ideal conditions for parasitoid larvae to develop within a pre-built refugium which could explain their relatively high parasitism rates in our study and others (e.g. Hrcek et al. 2013; Šigut et al. 2018). Conversely, cryptic caterpillars rely on camouflage and behavioural adaptations to evade detection by predators. However, they lack the chemical defences or protective structures necessary to avoid predation by vertebrate insectivores, such as birds, which can be the primary mediators of caterpillar populations in tropical regions (Mäntylä et al. 2011). This vulnerability makes cryptic caterpillars less suitable hosts for parasitoids, as they are more likely to be consumed after being parasitised, which may explain their reduced parasitism rate. Previous studies grouping caterpillars based on their feeding guilds concluded that semi-concealed feeders (shelter-building) have higher parasitism rates than exposed feeders (aposematic and cryptic) (e.g. Hrcek et al. 2013; Šigut et al. 2018). Our study reveals a more refined perspective on parasitism rates in caterpillars, highlighting that the presence of defensive traits in caterpillars may exert a more significant influence on parasitism rates than their feeding-guild.

Parasitism rates were extremely variable across strata for all caterpillars and between caterpillar defensive traits, which is likely due to the reduced incidence of parasitism when divided across all the forest strata. However, there is an apparent decrease in parasitism rates towards the upper canopy within all the defensive traits and across all caterpillars. In temperate forests, Chaij et al. (2016) found parasitism rates to be lowest in the upper canopy in concealed hosts and Šigut et al. (2018) found a similar pattern in leaf-chewing insects. One suggestion is that the increased structural complexity of adult tree crowns may lead to reduced foraging success for parasitoids (Godfray 1994; Yamazaki 2010). It is also probable that, similarly to their hosts, parasitoids avoid the upper canopy where the climatic conditions are less favourable. Adverse weather conditions such as increased wind speed and temperatures, which are more prevalent at the top of the canopy, have been shown to reduce the likelihood of parasitoids finding their hosts (Vosteen et al. 2020). Furthermore, caterpillars feeding on the less nutritious leaves in the upper canopy may have reduced fitness which would make them less suitable hosts for parasitoids. Another possibility is predation avoidance, parasitoids are susceptible to intraguild predation and Chmel et al. (2016) found that sallying, insectivorous birds were more abundant in higher vertical strata in a Cameroonian rainforest. As suggested by Šigut et al. (2018), parasitoids are more likely to aggregate in patches where they can minimise predator avoidance whilst increasing their likelihood of encountering a host. Additionally, this may explain why shelter-building caterpillars appear to occur at higher densities in the upper canopy but had the lowest parasitism rates, as they were occupying a more parasitoid-free space.

The higher peak is within the central midstory which is also where we found the highest diversity of caterpillars. Murdoch and Stewart-Oaten (1989) suggested that parasitoids may aggregate in patches with more potential hosts. For generalist parasitoids (those with multiple host species) especially, occupying the stratum with the highest diversity of parasitoids would maximise the likelihood of encountering a suitable host species. Additionally, this central midstory stratum could potentially represent the optimal combination of biotic (e.g. more caterpillar species and lower risk of predation) and abiotic (e.g. better visibility and wind protection) creating ideal foraging conditions for parasitoids, which would in turn, increase overall parasitism rates. Alternatively, these conditions might be more conducive for the hosts themselves, thereby explaining the observed high caterpillar diversity. In this scenario, the increased parasitoid activity could simply be a reflection of the conditions preferred by their hosts. Without more targeted studies, we can only speculate whether the observed patterns are primarily driven by the preferences and behaviours of the parasitoids, the hosts, or a complex interplay of both. Future research should aim to disentangle these possibilities to enhance our understanding of these intricate ecological dynamics. Curiously, cryptic and shelter-building caterpillars followed similar patterns in parasitism rates across strata, albeit with cryptic caterpillars being parasitised less frequently. This similarity may be attributed to their passive defence strategies of concealment and camouflage which both rely on avoiding detection to avoid predators and parasitoids. It is therefore possible that whilst shelter-building caterpillars are parasitised more frequently, the relative detection by parasitoids remains the same, leading to a similar pattern across the vertical forest gradient (Baer and Marquis 2020). For aposematic caterpillars, parasitism rates were notably higher in the upper midstory. Aposematic caterpillars rely on their warning signals and conspicuity to avoid predation, and previous studies have established that insectivores can identify and actively avoid aposematic insects (Exnerová et al. 2015; Aslam et al. 2020). Their conspicuity, and therefore predator protection, is likely enhanced in the upper midstory of the forest, where there is enhanced visibility from greater light penetration. However, this increased visibility also increases the likelihood of being detected by parasitoids that have been posited to be the most active in the midstory (Šigut et al. 2018). Therefore, it is possible that aposematic caterpillars occupying these strata are both easy to locate and less vulnerable to predation, making them ideal hosts for parasitoids. To our knowledge, this is the first study to compare parasitism rates in caterpillars across vertical forest strata in a tropical environment. Our findings suggest that the behaviour, morphology, and vertical distribution of caterpillars significantly impact their interactions with parasitoids. These results underscore the need for future research to further investigate the role of defensive traits, and vertical gradients in shaping caterpillar-parasitoid interaction in tropical forests.

## Conclusion

This study reveals the high diversity and complexity of caterpillar communities across a complete vertical gradient in a tropical forest in Cameroon. By dividing the forest into multiple vertical strata, we uncovered nuanced patterns of caterpillar diversity, density, specialisation, and parasitism rates that are obscured when comparing only the understory and canopy. We argue that categorising caterpillars by their defensive traits is a more intuitive approach than by their feeding guild when focussing on caterpillar-parasitoid interactions. The aposematic and shelter-building caterpillars had comparably high parasitism rates and should both be considered a “*safe haven”* for parasitoids. These results highlight the importance of the vertical dimensions of the forest and the natural history of caterpillars when studying their ecology in tropical forests and emphasises the importance of further research for unravelling the intricate and diverse factors that shape caterpillar communities and their interactions with parasitoids across entire vertical forest gradients.

### Supplementary Information

Below is the link to the electronic supplementary material.

Supplementary file1 (DOCX 148 KB)

## Data Availability

The data that support the findings of this study are available from the corresponding author upon reasonable request.

## References

[CR1] Abarca M, Boege K (2011). Fitness costs and benefits of shelter building and leaf trenching behaviour in a pyralid caterpillar. Ecol Entomol.

[CR2] Ashton LA, Nakamura A, Basset Y (2016). Vertical stratification of moths across elevation and latitude. J Biogeogr.

[CR3] Aslam M, Nedvěd O, Sam K (2020). Attacks by predators on artificial cryptic and aposematic insect larvae. Entomol Exp Appl.

[CR4] Baer CS, Marquis RJ (2020). Between predators and parasitoids: complex interactions among shelter traits, predation and parasitism in a shelter-building caterpillar community. Funct Ecol.

[CR5] Banašek-Richter C, Cattin M-F, Bersier L-F (2004). Sampling effects and the robustness of quantitative and qualitative food-web descriptors. J Theor Biol.

[CR6] Basset Y, Linsenmair KE, Davis AJ, Fiala B, Speight MR (2001). Invertebrates in the canopy of tropical rain forests How much do we really know?. Tropical forest canopies: ecology and management.

[CR7] Basset Y, Hammond PM, Barrios H (2003). Vertical stratification of arthropod assemblages. Arthropods of tropical forests.

[CR8] Bates D, Mächler M, Bolker B, Walker S (2014). Fitting linear mixed-effects models using lme4. J Stat Softw.

[CR9] Beck J, Holloway JD, Schwanghart W (2013). Undersampling and the measurement of beta diversity. Methods Ecol Evol.

[CR10] Blüthgen N, Menzel F, Blüthgen N (2006). Measuring specialization in species interaction networks. BMC Ecol.

[CR11] Bolker B (2017) Package ‘bbmle’. Tools for general maximum likelihood estimation. Tools for general maximum likelihood estimation. p 641

[CR12] Brehm G (2007). Contrasting patterns of vertical stratification in two moth families in a Costa Rican lowland rain forest. Basic Appl Ecol.

[CR13] Caro T, Ruxton G (2019). Aposematism: unpacking the defences. Trends Ecol Evol.

[CR14] Chaij J, Devoto M, Oleiro M (2016). Complexity of leaf miner–parasitoid food webs declines with canopy height in P atagonian beech forests. Ecol Entomol.

[CR15] Chao A, Jost L (2015). Estimating diversity and entropy profiles via discovery rates of new species. Methods Ecol Evol.

[CR16] Chen JM, Menges CH, Leblanc SG (2005). Global mapping of foliage clumping index using multi-angular satellite data. Remote Sens Environ.

[CR17] Chen W, Vasseur L, You M (2017). Parasitised caterpillars suffer reduced predation: potential implications for intra-guild predation. Sci Rep.

[CR18] Chmel K, Riegert J, Paul L, Novotný V (2016). Vertical stratification of an avian community in New Guinean tropical rainforest. Popul Ecol.

[CR19] Coelho MS, Carneiro MAA, Branco CA (2018). Species turnover drives β-diversity patterns across multiple spatial scales of plant-galling interactions in mountaintop grasslands. PLoS ONE.

[CR20] Coley PD, Barone JA (1996). Herbivory and plant defenses in tropical forests. Annu Rev Ecol Syst.

[CR21] Colwell R, Mao CX, Chang J (2004). Interpolating, extrapolating, and comparing incidence-based species accumulation curves. Ecology.

[CR22] Corff JL, Marquis RJ (1999). Differences between understorey and canopy in herbivore community composition and leaf quality for two oak species in Missouri. Ecol Entomol..

[CR23] Covarrubias-Camarillo T, Osorio-Beristain M, Legal L, Contreras-Garduño J (2016). *Baronia brevicornis* caterpillars build shelters to avoid predation. J Nat Hist.

[CR24] de Smedt P, Vangansbeke P, Bracke R (2019). Vertical stratification of moth communities in a deciduous forest in Belgium. Insect Conserv Divers.

[CR25] de Souza Amorim AD, Brown BV, Boscolo D (2022). Vertical stratification of insect abundance and species richness in an Amazonian tropical forest. Sci Rep.

[CR26] Delabye S, Rougerie R, Bayendi S (2019). Characterization and comparison of poorly known moth communities through DNA barcoding in two Afrotropical environments in Gabon. Genome.

[CR27] Dormann CF, Fründ J, Blüthgen N, Gruber B (2009). Indices, graphs and null models: analyzing bipartite ecological networks. Open Ecol J..

[CR28] Dyer LA, Gentry G (1999). Predicting natural-enemy responses to herbivores in natural and managed systems. Ecol Appl.

[CR29] Exnerová A, Ježová D, Štys P (2015). Different reactions to aposematic prey in 2 geographically distant populations of great tits. Behav Ecol.

[CR30] Frago E (2016). Interactions between parasitoids and higher order natural enemies: intraguild predation and hyperparasitoids. Curr Opin Insect Sci.

[CR31] Gentry GL, Dyer LA (2002). On the conditional nature of neotropical caterpillar defenses against their natural enemies. Ecology.

[CR32] Godfray HCJ (1994). Parasitoids: behavioral and evolutionary ecology.

[CR33] Greeney HF, Dyer LA, Smilanich AM (2012). Feeding by lepidopteran larvae is dangerous: a review of caterpillars’ chemical, physiological, morphological, and behavioral defenses against natural enemies. Invertebr Surviv J.

[CR34] Hausmann A, Diller J, Moriniere J (2020). DNA barcoding of fogged caterpillars in Peru: a novel approach for unveiling host-plant relationships of tropical moths (Insecta, Lepidoptera). PLoS ONE.

[CR35] Hawkins BA (1994). Pattern and process in host-parasitoid interactions.

[CR36] Hirao T, Murakami M, Kashizaki A (2009). Importance of the understory stratum to entomofaunal diversity in a temperate deciduous forest. Ecol Res.

[CR37] Horn HS (1966). Measurement of “overlap” in comparative ecological studies. Am Nat.

[CR38] Houska Tahadlova M, Mottl O, Jorge LR (2023). Trophic cascades in tropical rainforests: effects of vertebrate predator exclusion on arthropods and plants in Papua New Guinea. Biotropica.

[CR39] Hrcek J, Miller SE, Whitfield JB (2013). Parasitism rate, parasitoid community composition and host specificity on exposed and semi-concealed caterpillars from a tropical rainforest. Oecologia.

[CR40] Hsieh TC, Ma KH, Chao A, Hsieh MT (2016) Package ‘iNEXT’. interpolation and extrapolation for species diversity. https://www.chao/stat/nthu/edu/tw/blog/software-download/. Accessed 28 Feb 2017.

[CR41] Intachat J, Holloway JD (2000). Is there stratification in diversity or preferred flight height of geometroid moths in Malaysian lowland tropical forest?. Biodivers Conserv.

[CR42] Interian-Aguiñaga J, Parra-Tabla V, Abdala-Roberts L (2022). Effects of topical tree diversity and prey spatial distribution on predation by birds and arthropods. Arthropod Plant Interact.

[CR43] Kuznetsova A, Brockhoff PB, Christensen RH (2017). lmerTest package: tests in linear mixed effects models. J Stat Softw.

[CR44] Lampert EC, Dyer LA, Bowers MD (2010). Caterpillar chemical defense and parasitoid success: cotesia congregata parasitism of ceratomia catalpae. J Chem Ecol.

[CR45] Leles B, Xiao X, Pasion BO (2017). Does plant diversity increase top–down control of herbivorous insects in tropical forest?. Oikos.

[CR46] Lenth RV (2023) emmeans: estimated marginal means, aka least-squares means

[CR47] Lill JT, Marquis RJ, Ricklefs RE (2002). Host plants influence parasitism of forest caterpillars. Nature.

[CR48] Lim GS, Balke M, Meier R (2012). Determining species boundaries in a world full of rarity: singletons, species delimitation methods. Syst Biol.

[CR49] Mäntylä E, Klemola T, Laaksonen T (2011). Birds help plants: a meta-analysis of top-down trophic cascades caused by avian predators. Oecologia.

[CR50] Menken SB, Boomsma JJ, Van Nieukerken EJ (2010). Large-scale evolutionary patterns of host plant associations in the Lepidoptera. Evolution.

[CR51] Mitter C, Davis DR, Cummings MP (2017). Phylogeny and evolution of lepidoptera. Annu Rev Entomol.

[CR52] Moffett MW, Lowman M, Devy S, Ganesh T (2013). Comparative canopy biology and the structure of ecosystems. Treetops at risk.

[CR53] Molleman F, Walczak U, Melosik I (2022). What drives caterpillar guilds on a tree: enemy pressure, leaf or tree growth, genetic traits, or phylogenetic neighbourhood?. InSects.

[CR54] Morisita M (1959). Measuring of interspecific association and similarity between assemblages. Mem Fac Sci Kyushu Univ Ser E Biol.

[CR55] Murakami M, Yoshida K, Hara H, Toda MJ (2005). Spatio-temporal variation in Lepidopteran larval assemblages associated with oak, *Quercus*
*crispula*: the importance of leaf quality. Ecol Entomol.

[CR56] Murdoch WW, Stewart-Oaten A (1989). Aggregation by parasitoids and predators: effects on equilibrium and stability. Am Nat.

[CR57] Nakamura A, Kitching RL, Cao M (2017). Forests and their canopies: achievements and horizons in canopy science. Trends Ecol Evol.

[CR58] Oksanen J (2010) Vegan: community ecology package. https://www.vegan/r-forge/r-project.org/. Accessed 6 Nov 2023

[CR59] Parker GG, Brown MJ (2000). Forest canopy stratification—is it useful?. Am Nat.

[CR60] Pellissier L, Albouy C, Bascompte J (2018). Comparing species interaction networks along environmental gradients. Biol Rev.

[CR61] Pontes Ribeiro S, Basset Y (2007). Gall-forming and free-feeding herbivory along vertical gradients in a lowland tropical rainforest: the importance of leaf sclerophylly. Ecography.

[CR62] Posa MRC, Sodhi NS, Koh LP (2007). Predation on artificial nests and caterpillar models across a disturbance gradient in Subic Bay, Philippines. J Trop Ecol.

[CR63] R Development Core Team (2022). R: a language and environment for statistical computing.

[CR83] Rasband WS (2011) Imagej, us national institutes of health, Bethesda, Maryland, USA. http://imagej.nih.gov/ij/

[CR64] Ratnasingham S, Hebert PD (2013). A DNA-based registry for all animal species: the Barcode Index Number (BIN) system. PLoS ONE.

[CR65] Sam K, Koane B, Sam L (2020). Insect herbivory and herbivores of *Ficus* species along a rain forest elevational gradient in Papua New Guinea. Biotropica.

[CR66] Schowalter T, Chao J-T, Santos JC, Fernandes GW (2021). Canopy insect sampling. Measuring Arthropod Biodiversity.

[CR67] Schulze CH, Linsenmair KE, Fiedler K, Linsenmair KE, Davis AJ, Fiala B, Speight MR (2001). Understorey versus canopy: patterns of vertical stratification and diversity among Lepidoptera in a Bornean rain forest. Tropical forest canopies: ecology and management.

[CR68] Seifert CL, Lamarre GPA, Volf M (2020). Vertical stratification of a temperate forest caterpillar community in eastern North America. Oecologia.

[CR69] Seifert CL, Volf M, Jorge LR (2020). Plant phylogeny drives arboreal caterpillar assemblages across the Holarctic. Ecol Evol.

[CR70] Šigut M, Šigutová H, Šipoš J (2018). Vertical canopy gradient shaping the stratification of leaf-chewer–parasitoid interactions in a temperate forest. Ecol Evol.

[CR71] Singer MS, Clark RE, Lichter-Marck IH (2017). Predatory birds and ants partition caterpillar prey by body size and diet breadth. J Anim Ecol.

[CR72] Slinn HL, Richards LA, Dyer LA (2018). Across multiple species, phytochemical diversity and herbivore diet breadth have cascading effects on herbivore immunity and parasitism in a tropical model system. Front Plant Sci.

[CR73] Smilanich AM, Dyer LA, Chambers JQ, Bowers MD (2009). Immunological cost of chemical defence and the evolution of herbivore diet breadth. Ecol Lett.

[CR74] Stork NE, Grimbacher PS (2006). Beetle assemblages from an Australian tropical rainforest show that the canopy and the ground strata contribute equally to biodiversity. Proc R Soc B.

[CR75] Tvardikova K, Novotny V (2012). Predation on exposed and leaf-rolling artificial caterpillars in tropical forests of Papua New Guinea. J Trop Ecol.

[CR76] Ulyshen M (2011). Arthropod vertical stratification in temperate deciduous forests: implications for conservation-oriented management. Fuel Energy Abstr.

[CR77] Vázquez DP, Melián CJ, Williams NM (2007). Species abundance and asymmetric interaction strength in ecological networks. Oikos.

[CR78] Volf M, Klimeš P, Lamarre GP (2019). Quantitative assessment of plant-arthropod interactions in forest canopies: a plot-based approach. PLoS ONE.

[CR79] Vosteen I, Bianchi FJJA, Poelman EH (2020). Adverse weather conditions impede odor-guided foraging of parasitoids and reduce their host-finding success. Agr Ecosyst Environ.

[CR80] Walcroft AS, Brown KJ, Schuster WSF (2005). Radiative transfer and carbon assimilation in relation to canopy architecture, foliage area distribution and clumping in a mature temperate rainforest canopy in New Zealand. Agric for Meteorol.

[CR81] Wardhaugh CW (2014). The spatial and temporal distributions of arthropods in forest canopies: uniting disparate patterns with hypotheses for specialisation: drivers of insect distribution patterns. Biol Rev.

[CR82] Wilson JJ, Kress WJ, Erickson DL (2012). DNA Barcodes for Insects. DNA barcodes.

[CR84] Yamazaki K (2010). Leaf mines as visual defensive signals to herbivores. Oikos.

