# Supplementary material for: Vertical stratification and defensive traits of caterpillars against parasitoids in a lowland tropical forest in Cameroon
Source: Oecologia. 2024 Apr 13;204(4):915–30. doi: 10.1007/s00442-024-05542-x (PMC11062930; doi:10.1007/s00442-024-05542-x)
Supplement: Supplementary file 1 — Supplementary file1 (DOCX 148 KB) [file 442_2024_5542_MOESM1_ESM.docx]

# Supplementary Material

**Vertical stratification and defensive traits of caterpillars against parasitoids in a lowland tropical forest in Cameroon**

Sam Finnie*, Philip Butterill, Vojtech Novotny, Conor Redmond, Leonardo Ré Jorge, Tomokazu Abe, Greg P.A. Lamarre, Vincent Maicher, Katerina Sam

***Corresponding author:** Sam Finnie: [sam.c.finnie@gmail.com](mailto:sam.c.finnie@gmail.com)

**Appendix 1 Supplementary figures and tables**

**Table S1** Caterpillar abundance and species richness for each defensive trait (aposematic, cryptic, and shelter-building) for each stratum and across the entire vertical gradient (total).

| ***Stratum*** | ***Defensive traits*** | | | | | |
| --- | --- | --- | --- | --- | --- | --- |
|  | ***Aposematic*** | | ***Cryptic*** | | ***Shelter-building*** | |
|  | ***Abundance*** | ***Sp. Richness*** | ***Abundance*** | ***Sp. Richness*** | ***Abundance*** | ***Sp. Richness*** |
| **0-5m** | 25 | 12 | 27 | 16 | 34 | 10 |
| **5-10m** | 47 | 21 | 79 | 39 | 99 | 23 |
| **10-15m** | 65 | 23 | 98 | 39 | 155 | 25 |
| **15-20m** | 90 | 30 | 100 | 42 | 101 | 21 |
| **20-25m** | 64 | 23 | 100 | 38 | 66 | 13 |
| **25-30m** | 33 | 20 | 65 | 30 | 85 | 18 |
| **30-35m** | 23 | 14 | 35 | 19 | 51 | 14 |
| **35-40m** | 18 | 7 | 18 | 10 | 31 | 10 |
| **Total** | 365 | 66 | 522 | 109 | 622 | 73 |


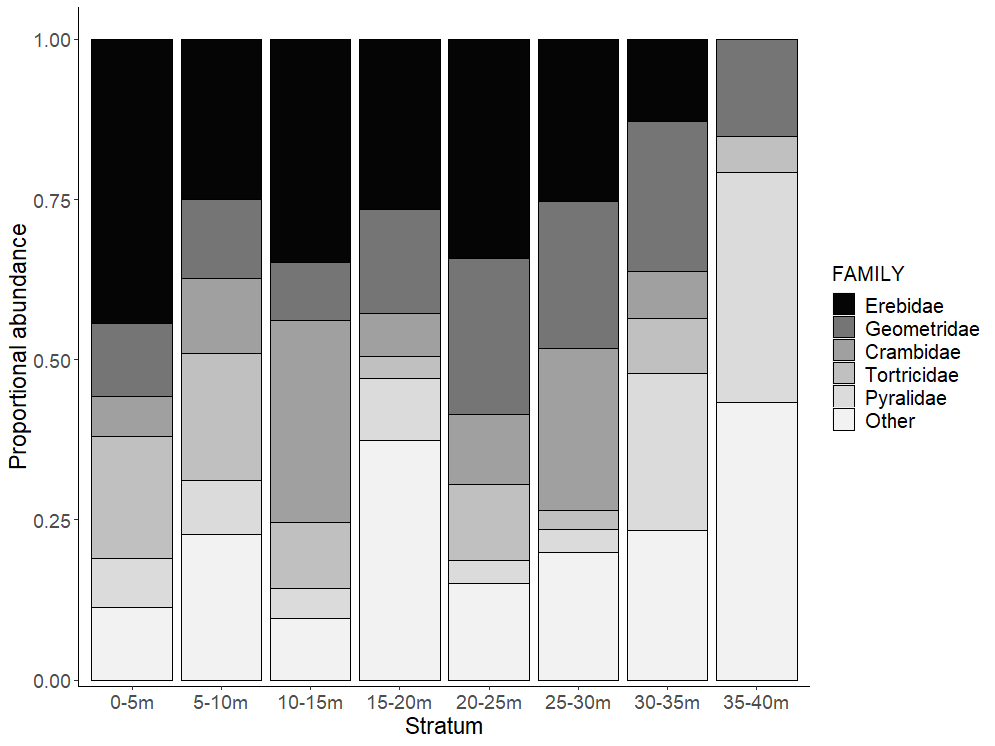
 **Fig. S1** The proportional abundance of the five most common caterpillar families (>100 individuals) and the remaining families combined (Other) for each forest stratum.

**Fig. S2** The prortional abundance of each of the caterpillar defensive traits for each forest stratum


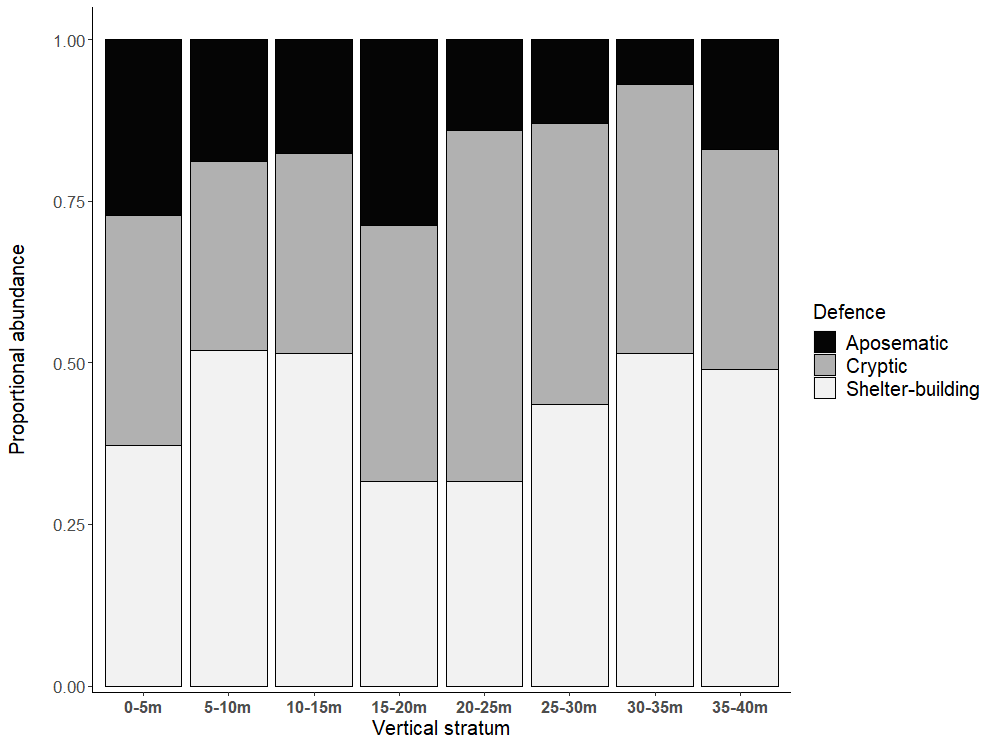


**Table S2** Metrics for caterpillar–host plant interaction networks of individual vertical strata. Based on comparisons with null models, Z-scores and respective P values are reported for weighted generality, weighted vulnerability, and weighted connectance

| *Stratum* | *Weighted specialisation networks metrics* | | | | | | | | |
| --- | --- | --- | --- | --- | --- | --- | --- | --- | --- |
|  | ***Generality*** | | | ***Vulnerability*** | | | ***Connectance*** | | |
|  | ***Obs.*** | ***Z-score*** | ***P value*** | ***Obs.*** | ***Z-score*** | ***P value*** | ***Obs.*** | ***Z-score*** | ***P value*** |
| 0-5m | 1.08 | 2.55 | <0.005 | 2.19 | 2.76 | <0.003 | 0.031 | 3.14 | <0.001 |
| 5-10m | 1.35 | 2.83 | <0.002 | 3.32 | 4.02 | <0.001 | 0.021 | 4.52 | <0.001 |
| 10-15m | 1.18 | 4.47 | <0.001 | 3.22 | 6.15 | <0.001 | 0.020 | 6.55 | <0.001 |
| 15-20m | 1.27 | 4.06 | <0.001 | 7.51 | 6.52 | <0.001 | 0.041 | 6.83 | <0.001 |
| 20-25m | 1.173 | 4.39 | <0.001 | 13.99 | 1.56 | <0.05 | 0.090 | 1.65 | <0.05 |
| 25-30m | 1.06 | 2.67 | <0.003 | 7.02 | 5.62 | <0.001 | 0.057 | 5.70 | <0.001 |
| 30-35m | 1.03 | 4.15 | <0.001 | 4.76 | 5.81 | <0.001 | 0.058 | 5.90 | <0.001 |
| 35-40m | 1 | 1.58 | 0.056 | 4.74 | 4.32 | <0.001 | 0.096 | 4.34 | <0.001 |

**Table S3** Total number of caterpillar species (and the percentage of the total species) found feeding on only one host plant species (i.e. specialist) and two or more species (i.e. generalist) for each defensive trait after the removal of singletons.

| **Defensive trait** | **Specialist** | **Generalist** |
| --- | --- | --- |
| **Aposematic** | 21 (45%) | 26 (55%) |
| **Cryptic** | 37 (46%) | 44 (54%) |
| **Shelter-building** | 31 (58%) | 20 (42%) |

**Table S4** Taxonomic checklist of caterpillar species included in this study as well as the total number of individuals for each species for each vertical stratum (0-5m, 5-10m, 10-15m, 15-20m, 20-25m, 25-30m, 30-35m, 35-40, and non-assigned, which are represented by 1, 2, 3, 4, 5, 6, 7, 8, and NA respectively) and across the entire vertical gradient (Total). The assigned defensive trait of each species: aposematic (A), cryptic (C), and Shelter-building (SB), and taxonomic BIN code is also given.

| *Taxonomy* | *Stratum* | | | | | | | | | *Total* | *Defence* | *BIN* |
| --- | --- | --- | --- | --- | --- | --- | --- | --- | --- | --- | --- | --- |
|  | ***1*** | ***2*** | ***3*** | ***4*** | ***5*** | ***6*** | ***7*** | ***8*** | ***NA*** |  |  |  |
| Crambidae |  |  |  |  |  |  |  |  |  |  |  |  |
| *Crambidae Sp1* | 4 | 11 | 77 | 17 | 18 | 39 | 6 |  | 17 | 189 | SB | BOLD:AEE1691 |
| *Crambidae Sp2* |  |  |  |  |  | 2 |  |  | 1 | 3 | SB | BOLD:AEE2153 |
| *Crambidae Sp3* | 1 | 7 | 9 | 1 |  |  |  |  |  | 18 | SB | BOLD:AEE7272 |
| *Crambidae Sp4* |  |  |  | 1 |  |  |  |  | 3 | 4 | SB | BOLD:AEI6464 |
| *Crambidae Sp5* |  | 3 | 1 |  |  |  |  |  |  | 4 | SB | BOLD:AEI6657 |
| *Crambidae Sp7* |  |  |  |  |  |  | 1 |  |  | 1 | SB | BOLD:AAZ8695 |
| *Crambidae Sp8* |  |  | 7 |  |  |  |  |  | 4 | 11 | SB | BOLD:ACW7425 |
| *Crambidae Sp9* |  |  |  |  | 3 | 1 |  |  |  | 4 | SB | BOLD:ADZ0948 |
| *Pardomima Sp1* |  | 4 |  |  |  |  |  |  |  | 4 | SB | BOLD:ABZ4988 |
| *Parotis Sp1* |  |  | 1 |  |  |  |  |  |  | 1 | SB | BOLD:AAL8871 |
| *Pyraustinae Sp1* |  |  |  |  |  |  |  |  | 1 | 1 | SB | BOLD:ACN8440 |
|  |  |  |  |  |  |  |  |  |  |  |  |  |
| Depressariidae |  |  |  |  |  |  |  |  |  |  |  |  |
| *Depressariidae Sp2* |  | 1 |  |  |  |  |  |  |  | 1 | C | BOLD:AEI7040 |
| *Depressariidae Sp3* |  |  |  | 1 | 1 |  |  |  |  | 2 | C | BOLD:ADZ1034 |
| *Depressariidae Sp4* | 1 |  |  | 7 | 2 |  |  |  | 2 | 12 | C | BOLD:AEN0449 |
|  |  |  |  |  |  |  |  |  |  |  |  |  |
| Ennominae |  |  |  |  |  |  |  |  |  |  |  |  |
| *Cleora Sp1* |  | 1 |  |  |  |  |  |  |  | 1 | C | BOLD:AAF4532 |
|  |  |  |  |  |  |  |  |  |  |  |  |  |
| Erebidae |  |  |  |  |  |  |  |  |  |  |  |  |
| *Achaea catocaloides* |  | 3 | 4 | 1 | 2 | 2 |  |  |  | 12 | C | BOLD:AAJ6790 |
| *Achaea Sp1* | 5 | 3 |  |  |  |  |  |  | 1 | 9 | C | BOLD:ACN5980 |
| *Amerila luteibarba* | 2 | 10 | 5 | 13 | 4 | 4 |  | 2 | 2 | 42 | A | BOLD:AAC9900 |
| *Amerila brunnea* |  | 1 | 3 |  | 1 | 1 |  |  |  | 6 | A | BOLD:AAH6190 |
| *Amerila niveivitrea* | 1 | 4 | 3 | 2 | 1 | 1 |  |  |  | 12 | A | BOLD:AAD8431 |
| *Amerila Sp1* | 1 |  |  |  | 1 |  |  |  |  | 2 | A | BOLD:ACF2883 |
| *Anoba Sp1* | 1 |  |  | 1 |  |  | 3 | 3 | 1 | 9 | C | BOLD:AEG2598 |
| *Anomis leona* |  |  |  | 1 |  |  |  |  | 1 | 2 | C | BOLD:AAG6096 |
| *Anomis Sp1* |  |  |  |  |  |  |  | 2 |  | 2 | C | BOLD:AEE4441 |
| *Arctiinae Sp1* |  |  |  | 3 |  |  |  |  | 2 | 5 | A | BOLD:AEN3282 |
| *Arctiinae Sp2* |  | 1 |  |  |  |  |  |  |  | 1 | A | BOLD:AEI5993 |
| *Arctiinae Sp3* |  | 1 |  |  |  |  |  |  |  | 1 | A | BOLD:AEI8572 |
| *Argyrostagma niobe* |  |  |  |  |  | 1 |  |  |  | 1 | A | BOLD:AAP1161 |
| *Balacra elegans* |  |  |  |  |  |  |  | 2 |  | 2 | A | BOLD:AAZ8679 |
| *Balacra flavimacula* | 1 |  | 12 | 2 | 1 |  |  |  |  | 16 | A | BOLD:AEE3340 |
| *Balacra haemalea* | 7 | 2 | 9 | 5 | 2 |  | 1 | 1 | 1 | 28 | A | BOLD:ACE6803 |
| *Balacra herona* | 5 |  | 3 | 2 |  | 2 | 1 | 1 |  | 14 | A | BOLD:AAZ8680 |
| *Cautatha drepanodes* |  |  |  | 1 |  |  |  |  |  | 1 | C | BOLD:AAL8493 |
| *Cerynea Sp1* |  | 5 | 4 | 3 | 9 | 3 |  |  |  | 24 | A | BOLD:AEE5739 |
| *Erebidae Sp1* |  |  | 3 |  |  |  |  |  |  | 3 | C | BOLD:AEE0461 |
| *Erebidae Sp10* |  |  |  |  | 1 |  | 1 |  |  | 2 | C | BOLD:AEI9658 |
| *Erebidae Sp11* |  |  |  | 2 |  |  |  |  |  | 2 | A | BOLD:ABW8960 |
| *Erebidae Sp15* | 3 | 2 |  |  |  |  |  |  |  | 5 | C | BOLD:AEI6009 |
| *Erebidae Sp2* | 3 | 1 |  |  | 1 |  |  |  |  | 5 | C | BOLD:AEE0597 |
| *Erebidae Sp3* |  |  | 1 |  |  |  |  |  |  | 1 | C | BOLD:AEE7367 |
| *Erebidae Sp4* |  | 1 |  |  | 2 | 3 |  |  |  | 6 | C | BOLD:ABW3624 |
| *Erebidae Sp5* |  |  |  |  |  |  | 1 | 2 | 4 | 7 | C | BOLD:ACN7658 |
| *Erebidae Sp6* |  | 4 |  |  |  |  |  |  |  | 4 | A | BOLD:ACR1238 |
| *Erebidae Sp7* |  |  | 1 |  |  |  |  | 1 |  | 2 | C | BOLD:ACR1238 |
| *Erebidae Sp8* |  | 1 | 1 |  | 1 | 2 |  |  |  | 5 | C | BOLD:AEE9494 |
| *Erebidae Sp9* |  |  | 3 | 1 | 1 | 2 |  |  | 1 | 8 | A | BOLD:AEI4753 |
| *Erebidae Sp12* |  | 1 | 2 |  |  |  |  |  |  | 3 | C | BOLD:AEE8644 |
| *Erebidae Sp13* | 1 |  |  |  |  |  |  |  |  | 1 | C | BOLD:AEI4420 |
| *Erebidae Sp14* |  |  |  |  |  |  |  |  | 1 | 1 | C | BOLD:AEI5880 |
| *Erebidae Sp16* |  |  |  |  |  |  | 1 |  |  | 1 | C | BOLD:AEI3886 |
| *Eublemma Sp1* |  |  |  |  | 1 | 2 |  |  |  | 3 | SB | BOLD:AEE8923 |
| *Euproctis Sp1* |  | 1 |  | 1 |  | 2 |  | 1 |  | 5 | A | BOLD:AAL8759 |
| *Euproctis Sp2* |  |  | 1 |  |  |  |  |  |  | 1 | A | BOLD:AAZ8438 |
| *Hippurarctia Sp1* | 1 |  |  |  |  |  |  |  |  | 1 | C | BOLD:AEH2268 |
| *Lymantriinae Sp2* |  |  |  |  | 1 | 1 |  |  |  | 2 | A | BOLD:AEE2466 |
| *Lymantriinae Sp3* |  |  | 1 | 3 |  | 1 | 1 |  |  | 6 | A | BOLD:AEI6191 |
| *Lymantriinae Sp1* |  | 1 |  | 2 | 1 |  |  |  |  | 4 | A | BOLD:AAH6293 |
| *Lymantriinae Sp4* |  |  | 1 |  |  |  |  | 1 |  | 2 | A | BOLD:ACS1265 |
| *Manulea Sp1* | 2 |  | 1 |  |  |  | 1 | 1 | 2 | 7 | A | BOLD:AAG6346 |
| *Marcipa Sp1* |  |  | 1 |  |  |  | 1 |  | 1 | 3 | A | BOLD:AEE9713 |
| *Melisa diptera* |  |  |  | 1 |  |  |  |  |  | 1 | A | BOLD:AAZ8922 |
| *Oruza Sp1* |  |  |  |  |  | 1 |  |  | 1 | 2 | C | BOLD:AEE4870 |
| *Paremonia Sp1* |  |  | 1 | 1 | 1 |  |  |  |  | 3 | A | BOLD:AEE8835 |
| *Plecoptera Sp1* |  | 6 | 40 | 1 | 1 | 1 |  |  |  | 49 | C | BOLD:ACN8511 |
| *Rhipidarctia Sp1* |  | 1 |  |  |  |  | 1 |  |  | 2 | A | BOLD:ACN4127 |
| *Rougeotiana Sp1* | 1 | 1 | 3 | 16 | 31 | 11 |  |  | 1 | 64 | C | BOLD:AAL8616 |
| *Rougeotiana Sp2* | 1 | 1 | 2 | 3 | 3 | 2 |  |  |  | 12 | C | BOLD:AAH6227 |
| *Soloe trigutta* |  | 2 |  |  |  |  |  |  |  | 2 | C | BOLD:AAC6175 |
| *Tumicla Sp1* |  |  |  | 10 |  |  |  |  |  | 10 | A | BOLD:ACW5918 |
|  |  |  |  |  |  |  |  |  |  |  |  |  |
| Euteliidae |  |  |  |  |  |  |  |  |  |  |  |  |
| *Eutelia leucodelta* |  |  |  |  | 1 |  |  |  |  | 1 | C | BOLD:AEJ3716 |
| *Euteliidae Sp1* |  |  |  | 3 | 5 | 5 |  |  |  | 13 | A | BOLD:AEN2119 |
|  |  |  |  |  |  |  |  |  |  |  |  |  |
| Gelechiidae |  |  |  |  |  |  |  |  |  |  |  |  |
| *Gelechiidae Sp1* |  |  |  |  |  |  | 1 | 1 | 1 | 3 | SB | BOLD:ADG9652 |
| *Gelechiidae Sp10* |  | 2 |  |  |  |  |  |  |  | 2 | SB | BOLD:AEI9210 |
| *Gelechiidae Sp12* |  |  |  |  |  | 1 |  | 1 |  | 2 | SB | BOLD:ADH3603 |
| *Gelechiidae Sp3* |  |  |  |  |  | 3 | 3 |  |  | 6 | SB | BOLD:AEH9388 |
| *Gelechiidae Sp4* |  |  |  |  | 1 | 6 | 2 | 1 | 3 | 13 | SB | BOLD:AEE6047 |
| *Gelechiidae Sp5* |  | 1 |  |  |  |  |  |  |  | 1 | SB | BOLD:AEE6345 |
| *Gelechiidae Sp6* |  |  | 1 | 1 |  | 1 |  |  |  | 3 | SB | BOLD:AEI0060 |
| *Gelechiidae Sp7* |  |  |  |  |  |  | 1 |  |  | 1 | SB | BOLD:AEI3356 |
| *Gelechiidae Sp8* | 1 | 2 |  |  |  |  |  |  |  | 3 | SB | BOLD:AEI8015 |
| *Gelechiidae Sp9* |  |  |  |  |  | 1 |  |  |  | 1 | SB | BOLD:AEI8048 |
| *Gelechiidae Sp11* |  |  |  |  |  |  |  | 1 |  | 1 | SB | BOLD:AEI8085 |
| *Gelechiidae Sp13* |  |  |  | 1 |  | 1 |  |  | 1 | 3 | SB | BOLD:ADJ2439 |
| *Gelechiidae Sp14* |  |  |  | 1 |  |  |  |  | 1 | 2 | SB | BOLD:ADY9887 |
| *Gelechiidae Sp15* |  | 3 | 2 |  |  |  |  |  | 1 | 6 | SB | BOLD:AEE9495 |
| *Gelechiidae Sp16* |  | 1 |  |  |  | 1 |  |  |  | 2 | SB | BOLD:AEH9345 |
| *Gelechiidae Sp17* |  |  |  |  |  |  |  | 1 | 1 | 2 | SB | BOLD:AEI0814 |
|  |  |  |  |  |  |  |  |  |  |  |  |  |
| Geometridae |  |  |  |  |  |  |  |  |  |  |  |  |
| *Antharmostes Sp1* |  |  |  |  |  |  |  |  | 1 | 1 | C | BOLD:AAL8485 |
| *Antharmostes Sp2* |  |  | 1 |  | 1 |  |  |  |  | 2 | C | BOLD:AEI8982 |
| *Buzura Sp1* |  | 1 |  |  |  |  |  |  |  | 1 | C | BOLD:AEI8982 |
| *Chelotephrina Sp1* |  |  |  |  | 1 |  |  |  |  | 1 | C | BOLD:AEF0041 |
| *Cleora dargei* | 1 | 5 | 2 | 3 |  |  |  |  |  | 11 | C | BOLD:AAI4131 |
| *Cleora lamottei* |  | 2 | 2 | 1 | 2 | 2 | 1 |  | 2 | 12 | C | BOLD:ABY6002 |
| *Cleora oculata* |  |  |  | 1 |  |  | 1 | 1 |  | 3 | C | BOLD:AAD7512 |
| *Colocleora linearis* |  | 1 | 2 | 1 | 2 |  |  |  | 1 | 7 | C | BOLD:AAH6357 |
| *Colocleora divisaria* |  |  | 2 | 3 | 3 | 7 | 2 |  | 1 | 18 | C | BOLD:AAF9551 |
| *Colocleora indivisa* |  |  |  |  | 1 |  |  |  |  | 1 | C | BOLD:AAF9539 |
| *Cyclophora diplosticta* | 1 |  |  |  |  | 6 | 1 |  | 1 | 9 | C | BOLD:AAF9525 |
| *Cyclophora sp2* |  |  | 2 |  |  |  |  |  |  | 2 | C | BOLD:AAV7876 |
| *Dasymacaria nr. plebeia* |  |  | 1 |  | 1 | 1 |  |  |  | 3 | C | BOLD:ACN8806 |
| *Dasymacaria plebeia* |  |  |  |  |  |  | 1 |  | 1 | 2 | C | BOLD:ABA8685 |
| *Dioptrochasma specularia* |  |  | 1 |  |  |  |  |  | 1 | 2 | C | BOLD:ACM8628 |
| *Dorsifulcrum canui* |  |  |  | 1 | 1 |  |  |  |  | 2 | C | BOLD:AAL8852 |
| *Dorsifulcrum Sp1* |  | 1 |  | 1 |  |  |  |  |  | 2 | C | BOLD:AAH6418 |
| *Ennominae Sp1* |  |  | 1 | 6 | 2 |  |  |  | 1 | 10 | C | BOLD:AEN4459 |
| *Ennominae Sp2* |  |  | 1 |  |  |  |  |  |  | 1 | C | BOLD:AAP1549 |
| *Ennominae Sp3* |  | 3 | 1 | 1 |  |  |  |  |  | 5 | C | BOLD:ADH4832 |
| *Eulycia Sp1* |  | 1 |  |  | 3 | 3 | 7 | 4 | 6 | 24 | C | BOLD:AAQ1712 |
| *Eupithecia Sp1* |  |  |  |  |  | 1 |  |  |  | 1 | C | BOLD:AEI0703 |
| *Gelasmodes fasciata* |  |  |  |  |  |  |  |  | 1 | 1 | C | BOLD:AAL8839 |
| *Geolyces Geolyces nr. contenta* |  |  |  |  |  |  |  |  | 2 | 2 | C | BOLD:AAH6123 |
| *Geolyces smithi* | 1 | 2 | 2 | 3 | 2 |  |  |  |  | 10 | C | BOLD:AAV3418 |
| *Geolyces Sp2* | 2 |  | 2 | 1 |  | 1 |  |  |  | 6 | C | BOLD:AAF9540 |
| *Geolyces Sp3* |  |  |  |  |  | 1 |  |  |  | 1 | C | BOLD:ACN5336 |
| *Geolyces Sp4* |  |  |  | 1 | 1 | 2 |  |  |  | 4 | C | BOLD:ACN7692 |
| *Geometridae Sp1* |  | 2 | 2 |  |  |  |  |  | 1 | 5 | C | BOLD:AEE2500 |
| *Geometridae Sp2* |  |  |  |  |  | 1 |  |  |  | 1 | C | BOLD:ACN3388 |
| *Megadrepana cinerea* |  |  |  | 2 |  |  |  |  |  | 2 | C | BOLD:AAH6217 |
| *Metallochlora misera* |  |  | 2 | 3 | 1 |  |  |  |  | 6 | C | BOLD:AAU3523 |
| *Metallochlora Sp1* |  |  |  |  | 5 | 1 |  |  |  | 6 | C | BOLD:AAU0078 |
| *Metallospora catori* |  |  |  |  |  |  | 1 | 1 |  | 2 | C | BOLD:AAP2330 |
| *Miantochora sp1* |  |  |  |  | 1 | 1 |  |  |  | 2 | C | BOLD:AAF9523 |
| *Miantochora sp2* |  | 1 | 1 |  |  |  |  |  |  | 2 | C | BOLD:ABZ0934 |
| *Miantochora venerata* |  |  |  |  | 1 |  |  |  |  | 1 | C | BOLD:AAH6366 |
| *Omphalucha Sp1* |  |  |  | 1 |  |  |  |  |  | 1 | C | BOLD:ACE9415 |
| *Prasinocyma Sp1* |  |  |  |  | 1 | 1 |  |  |  | 2 | C | BOLD:AAF8220 |
| *Prasinocyma Sp2* |  |  |  |  |  | 1 | 1 |  | 1 | 3 | C | BOLD:ADB3411 |
| *Pycnostega stilbia* |  |  |  | 1 | 2 | 1 | 1 |  |  | 5 | C | BOLD:AAP3132 |
| *Racotis squalida* | 3 | 1 |  |  |  |  |  |  |  | 4 | C | BOLD:AAL0856 |
| *Racotis zebrina* |  | 1 | 1 |  |  | 4 |  |  |  | 6 | C | BOLD:AAF9530 |
| *Thalassodes immissaria* | 1 |  |  | 2 | 4 | 2 | 5 | 2 | 3 | 19 | C | BOLD:AAH6372 |
| *Thenopa diversa* |  | 3 |  | 2 | 1 |  |  |  |  | 6 | C | BOLD:ACR1171 |
| *Xylopteryx Sp1* |  | 2 |  |  |  |  |  |  |  | 2 | C | BOLD:ACG8725 |
| *Zamarada emaciata* |  |  | 1 | 12 | 11 | 2 | 1 |  | 2 | 29 | C | BOLD:AAL9687 |
|  |  |  |  |  |  |  |  |  |  |  |  |  |
| Gracillariidae |  |  |  |  |  |  |  |  |  |  |  |  |
| *Caloptilia sapporella* |  |  |  |  | 1 | 1 |  |  |  | 2 | SB | BOLD:AEF5553 |
| *Gracillariidae sp1* |  |  |  |  | 1 |  |  |  |  | 1 | SB | BOLD:AEO4471 |
| *Lamprolectica Sp1* |  |  |  |  |  | 4 |  |  |  | 4 | SB | BOLD:AAG7423 |
|  |  |  |  |  |  |  |  |  |  |  |  |  |
| Hesperiidae |  |  |  |  |  |  |  |  |  |  |  |  |
| *Fresna nyassae* |  |  |  | 1 |  |  |  |  |  | 1 | C | BOLD:ADJ8751 |
|  |  |  |  |  |  |  |  |  |  |  |  |  |
| Immidae |  |  |  |  |  |  |  |  |  |  |  |  |
| *Immidae Sp1* |  |  |  |  |  | 1 |  |  | 1 | 2 | C | BOLD:AEI0061 |
|  |  |  |  |  |  |  |  |  |  |  |  |  |
| Lasiocampidae |  |  |  |  |  |  |  |  |  |  |  |  |
| *Gonobombyx angulata* | 1 | 1 | 1 |  |  |  |  |  |  | 3 | A | BOLD:AAH6137 |
| *Lasiocampidae Sp1* | 1 |  |  |  |  |  |  |  |  | 1 | A | BOLD:AAW0452 |
| *Leipoxais proboscifera* |  |  |  | 1 |  |  |  |  |  | 1 | A | BOLD:AAV7792 |
| *Pachymetana Sp1* |  |  |  | 1 | 1 |  |  |  |  | 2 | A | BOLD:AEE9328 |
| *Pachymetana Sp2* |  |  |  |  |  |  |  |  | 1 | 1 | A | BOLD:AEE2135 |
| *Pachytrina Sp1* |  | 1 |  |  |  |  |  |  |  | 1 | A | BOLD:AEN3023 |
| *Ptyssophlebia discocellularis* |  |  |  |  |  |  | 2 |  |  | 2 | A | BOLD:AAL8695 |
| *Stoermeriana Sp1* |  | 1 |  |  |  | 1 | 1 |  |  | 3 | A | BOLD:AAL9924 |
|  |  |  |  |  |  |  |  |  |  |  |  |  |
| Limacodidae |  |  |  |  |  |  |  |  |  |  |  |  |
| *Anilina Sp1* |  |  | 1 | 1 |  |  | 1 |  |  | 3 | A | BOLD:AEE4541 |
| *Delorhachis Sp1* |  |  |  |  |  | 1 |  |  |  | 1 | A | BOLD:AEI2806 |
| *Latoia Sp1* |  | 1 |  |  |  |  | 4 |  |  | 5 | A | BOLD:AEK7653 |
| *Limacodidae Sp1* |  |  |  | 1 | 1 |  | 1 |  |  | 3 | A | BOLD:AEH8965 |
| *Limacodidae Sp2* |  |  |  |  | 10 |  | 1 | 10 |  | 21 | A | BOLD:AEI7405 |
| *Limacodidae Sp3* |  |  |  |  |  | 1 |  |  |  | 1 | A | BOLD:AEE9784 |
| *Limacodidae Sp4* |  | 1 | 1 |  | 2 | 1 | 1 |  |  | 6 | A | BOLD:ABV2905 |
| *Limacodidae Sp5* |  |  |  |  |  | 1 |  |  |  | 1 | A | BOLD:AEE3799 |
| *Limacodidae Sp6* |  |  | 1 | 1 | 1 |  |  |  | 1 | 4 | A | BOLD:AAE9783 |
| *Limacodidae Sp7* |  |  |  | 1 |  |  |  |  |  | 1 | A | BOLD:AEI6891 |
| *Limacodidae Sp8* |  |  | 3 |  | 11 | 1 |  |  | 1 | 16 | A | BOLD:AEI7009 |
| *Limacodinae Sp1* |  |  |  |  |  |  | 1 |  |  | 1 | A | BOLD:ACR1185 |
| *Trachyptena Sp1* |  |  |  |  |  |  |  |  | 1 | 1 | A | BOLD:AEE8473 |
|  |  |  |  |  |  |  |  |  |  |  |  |  |
| Lycaenidae |  |  |  |  |  |  |  |  |  |  |  |  |
| *Aphnaeus argyrocyclus* |  |  |  |  |  | 1 |  |  |  | 1 | C | BOLD:AAQ3171 |
| *Epitolina dispar* |  | 1 |  |  |  |  |  |  |  | 1 | A | BOLD:AAI5315 |
| *Lycaenidae Sp1* |  |  |  |  | 1 |  |  | 1 |  | 2 | C | BOLD:AEE1211 |
| *Lycaenidae Sp2* | 1 |  |  | 1 |  |  |  |  |  | 2 | A | BOLD:AEN8829 |
| *Ornipholidotos Sp1* | 1 |  |  |  |  |  |  |  |  | 1 | A | BOLD:ABY9895 |
| *Syrmoptera Sp2* | 1 |  | 1 |  | 1 |  |  |  |  | 3 | A | BOLD:AAH7454 |
|  |  |  |  |  |  |  |  |  |  |  |  |  |
| Metarbelidae |  |  |  |  |  |  |  |  |  |  |  |  |
| *Metarbelidae Sp1* | 1 |  |  |  |  |  |  |  |  | 1 | SB | BOLD:ABV0335 |
|  |  |  |  |  |  |  |  |  |  |  |  |  |
| Noctuidae |  |  |  |  |  |  |  |  |  |  |  |  |
| *Amphipyrinae Sp1* |  |  |  | 1 |  |  |  |  |  | 1 | C | BOLD:AAH6007 |
| *Lophoptera Sp1* |  |  |  | 1 | 3 | 1 |  |  | 1 | 6 | A | BOLD:AED4227 |
| *Noctuidae Sp1* |  | 1 |  |  |  |  |  |  |  | 1 | C | BOLD:AEI4967 |
| *Noctuidae Sp2* |  | 3 |  |  |  |  |  |  |  | 3 | C | BOLD:AEI6104 |
| *Noctuidae Sp3* |  |  |  |  |  | 1 |  |  |  | 1 | A | BOLD:AEO2444 |
| *Noctuidae Sp10* |  |  | 1 |  |  |  | 1 |  |  | 2 | C | BOLD:ACN6535 |
| *Noctuidae Sp11* |  |  |  |  | 1 |  |  |  |  | 1 | C | BOLD:AEI3801 |
| *Noctuidae Sp4* |  |  | 1 |  |  |  |  |  |  | 1 | C | BOLD:AAH6400 |
| *Noctuidae Sp5* |  |  |  |  |  |  | 3 |  | 1 | 4 | C | BOLD:AAL8519 |
| *Noctuidae Sp6* |  |  | 1 |  |  |  |  |  |  | 1 | C | BOLD:AAL8660 |
| *Noctuidae Sp7* |  |  |  | 1 |  |  |  |  |  | 1 | C | BOLD:AAY7143 |
| *Noctuidae Sp8* |  | 1 |  |  |  |  |  |  |  | 1 | C | BOLD:ACN6341 |
| *Noctuidae Sp9* |  |  | 6 | 13 | 3 |  |  |  |  | 22 | A | BOLD:ACQ1007 |
|  |  |  |  |  |  |  |  |  |  |  |  |  |
| Nolidae |  |  |  |  |  |  |  |  |  |  |  |  |
| *Lophocrama phoennicochlora* |  |  |  |  |  |  | 2 |  |  | 2 | C | BOLD:AAD9056 |
| *Meganola Sp1* |  |  | 1 | 2 |  |  |  |  | 1 | 4 | A | BOLD:AAP3232 |
| *Negeta approximans* |  | 2 |  | 2 |  |  |  |  |  | 4 | C | BOLD:ACR3594 |
| *Nolidae Sp1* |  |  |  | 26 |  |  |  |  |  | 26 | C | BOLD:AEE4029 |
| *Nolidae Sp2* |  |  | 1 | 1 |  |  |  |  |  | 2 | A | BOLD:AEO3954 |
|  |  |  |  |  |  |  |  |  |  |  |  |  |
| Notodontidae |  |  |  |  |  |  |  |  |  |  |  |  |
| *Anaphe venata* |  |  |  | 10 |  |  |  |  |  | 10 | A | BOLD:AEI3868 |
| *Daulopaectes Sp1* |  |  |  | 1 |  |  |  |  |  | 1 | C | BOLD:AEF0256 |
| *Desmeocraera Sp1* |  |  |  | 1 |  |  |  |  |  | 1 | C | BOLD:ACN6989 |
| *Janthinisca Sp1* |  |  |  | 1 |  | 1 |  |  |  | 2 | A | BOLD:AAL8882 |
| *Notodontidae Sp1* |  |  | 1 | 1 |  |  |  |  |  | 2 | C | BOLD:AEE9010 |
| *Notodontidae Sp2* |  | 1 | 2 | 6 |  |  |  |  | 1 | 10 | C | BOLD:AEI4093 |
| *Notodontidae Sp3* |  |  |  | 1 |  |  |  |  |  | 1 | C | BOLD:AEI8602 |
| *Notodontidae Sp4* |  |  | 2 | 1 |  |  |  |  | 1 | 4 | C | BOLD:AAH6298 |
| *Notodontidae Sp5* |  | 2 | 1 |  |  |  |  |  |  | 3 | C | BOLD:AAH6387 |
| *Notodontidae Sp6* |  |  | 1 |  |  |  |  |  |  | 1 | C | BOLD:AAL8512 |
| *Peratodonta Sp1* |  |  |  |  | 1 | 1 |  |  |  | 2 | C | BOLD:AAL8504 |
|  |  |  |  |  |  |  |  |  |  |  |  |  |
| Nymphalidae |  |  |  |  |  |  |  |  |  |  |  |  |
| *Bebearia Sp1* |  | 1 |  |  |  |  |  |  |  | 1 | A | BOLD:AAC0918 |
| *Catuna Sp1* |  |  |  | 10 |  |  |  |  |  | 10 | A | BOLD:AEE5128 |
| *Charaxes Sp1* | 1 | 1 | 1 | 2 | 1 | 1 |  |  | 3 | 10 | C | BOLD:AAA6009 |
| *Charaxes Sp2* |  | 1 | 1 | 3 | 1 |  |  |  |  | 6 | C | BOLD:AAC1064 |
| *Charaxes Sp3* |  |  |  |  | 1 |  |  |  |  | 1 | C | BOLD:AAC1745 |
| *Euphaedra Sp1* |  |  |  | 1 |  |  |  |  |  | 1 | A | BOLD:AAB0368 |
| *Euptera neptunoides* |  |  |  | 1 |  |  |  |  |  | 1 | A | BOLD:ACJ5855 |
| *Euryphura Sp1* |  |  |  |  | 1 |  |  |  |  | 1 | A | BOLD:AAA7865 |
| *Libythea labdaca* |  | 3 | 1 |  |  |  |  |  |  | 4 | C | BOLD:AAY7212 |
|  |  |  |  |  |  |  |  |  |  |  |  |  |
| Papilionidae |  |  |  |  |  |  |  |  |  |  |  |  |
| *Graphium policenes* |  | 3 |  |  |  |  |  |  | 1 | 4 | A | BOLD:AAF7125 |
|  |  |  |  |  |  |  |  |  |  |  |  |  |
| Pieridae |  |  |  |  |  |  |  |  |  |  |  |  |
| *Pieridae Sp1* |  |  |  | 1 |  |  |  |  |  | 1 | SB | BOLD:ACN8780 |
|  |  |  |  |  |  |  |  |  |  |  |  |  |
| Pterophoridae |  |  |  |  |  |  |  |  |  |  |  |  |
| *Stenoptilia Sp1* |  | 2 | 1 |  |  |  |  |  |  | 3 | SB | BOLD:AEE9782 |
|  |  |  |  |  |  |  |  |  |  |  |  |  |
|  |  |  |  |  |  |  |  |  |  |  |  |  |
| Pyralidae |  |  |  |  |  |  |  |  |  |  |  |  |
| *Mussidia Sp1* |  |  | 1 |  |  |  |  |  |  | 1 | SB | BOLD:AAM7055 |
| *Pyralidae Sp1* |  |  | 1 |  |  |  |  |  |  | 1 | SB | BOLD:AEI5889 |
| *Pyralidae Sp2* |  | 9 | 1 | 1 | 3 | 2 | 1 |  | 2 | 19 | SB | BOLD:AAH5937 |
| *Pyralidae Sp5* |  |  |  | 7 |  |  |  |  |  | 7 | SB | BOLD:AEE6695 |
| *Pyralidae Sp6* | 6 | 7 | 1 |  |  |  |  |  |  | 14 | SB | BOLD:AAE9011 |
| *Pyralidae Sp7* |  |  |  | 1 |  |  |  |  |  | 1 | SB | BOLD:AEI6192 |
| *Pyralidae Sp3* |  |  |  | 3 |  | 3 | 22 | 19 | 3 | 50 | SB | BOLD:AAL8720 |
| *Pyralidae Sp4* |  | 2 | 10 | 15 | 4 | 1 |  |  | 1 | 33 | SB | BOLD:ADJ2942 |
|  |  |  |  |  |  |  |  |  |  |  |  |  |
| Saturniidae |  |  |  |  |  |  |  |  |  |  |  |  |
| *Carnegia mirabilis* |  |  |  |  | 2 |  |  |  |  | 2 | A | BOLD:AAA7819 |
| *Carnegia Sp1* |  |  |  |  |  | 1 | 6 |  |  | 7 | A | BOLD:AEE2751 |
| Pselaphelia neglecta |  | 5 | 1 |  |  |  |  |  |  | 6 | A | BOLD:AAB1026 |
|  |  |  |  |  |  |  |  |  |  |  |  |  |
| Sphingidae |  |  |  |  |  |  |  |  |  |  |  |  |
| *Nephele rosae* |  | 1 | 1 |  |  |  |  |  |  | 2 | C | BOLD:ACE9121 |
| *Polyptychus carteri* |  | 2 |  | 1 |  |  |  |  |  | 3 | C | BOLD:AAA9585 |
|  |  |  |  |  |  |  |  |  |  |  |  |  |
| Tineidae |  |  |  |  |  |  |  |  |  |  |  |  |
| *Tineidae Sp1* |  | 3 |  |  |  |  |  |  |  | 3 | SB | BOLD:AEE2426 |
| *Tineidae Sp2* |  | 1 |  |  |  |  |  |  |  | 1 | SB | BOLD:AEE9452 |
|  |  |  |  |  |  |  |  |  |  |  |  |  |
| Tortricidae | + |  |  |  |  |  |  |  |  |  |  |  |
| *Afroploce Sp1* |  |  | 2 | 2 | 12 | 5 | 1 |  | 3 | 25 | SB | BOLD:AAJ2244 |
| *Choristoneura dinota* |  |  | 2 |  | 4 |  |  |  |  | 6 | SB | BOLD:AAI0649 |
| *Eccopsis Sp1* |  |  | 2 |  |  |  |  |  |  | 2 | SB | BOLD:ACN7373 |
| *Eccopsis Sp2* |  |  |  |  |  |  | 1 | 2 |  | 3 | SB | BOLD:ABV2389 |
| *Tortricidae Sp1* |  |  |  |  |  |  | 1 |  |  | 1 | SB | BOLD:AEF0103 |
| *Tortricidae Sp10* |  | 1 |  |  |  |  |  |  |  | 1 | SB | BOLD:AEE9625 |
| *Tortricidae Sp11* |  |  |  | 2 |  |  |  |  |  | 2 | SB | BOLD:AEI0984 |
| *Tortricidae Sp12* |  |  | 1 |  |  |  |  |  | 1 | 2 | SB | BOLD:AEI1685 |
| *Tortricidae Sp13* |  |  |  |  |  |  | 1 |  |  | 1 | SB | BOLD:AEM7284 |
| *Tortricidae Sp15* |  |  |  | 1 |  |  |  |  |  | 1 | SB | BOLD:AEN3931 |
| *Tortricidae Sp16* |  |  |  |  |  |  |  |  | 1 | 1 | SB | BOLD:AEI4990 |
| *Tortricidae Sp17* |  |  | 1 |  |  |  |  |  |  | 1 | SB | BOLD:AEI5133 |
| *Tortricidae Sp18* |  |  |  | 2 |  |  |  |  |  | 2 | SB | BOLD:AEM6824 |
| *Tortricidae Sp2* |  |  |  |  | 3 |  | 4 | 1 |  | 8 | SB | BOLD:AEF0154 |
| *Tortricidae Sp20* | 7 | 17 |  |  |  |  |  |  |  | 24 | SB | BOLD:AEO0854 |
| *Tortricidae Sp3* |  |  | 2 |  | 1 |  |  |  |  | 3 | SB | BOLD:AEH0539 |
| *Tortricidae Sp4* |  | 1 |  |  |  |  |  |  |  | 1 | SB | BOLD:AEH1664 |
| *Tortricidae Sp5* |  | 4 | 1 |  |  |  |  |  |  | 5 | SB | BOLD:AEH9321 |
| *Tortricidae Sp6* |  |  | 3 |  |  |  |  |  |  | 3 | SB | BOLD:AEM5086 |
| *Tortricidae Sp7* | 6 | 13 |  |  |  |  |  |  | 1 | 20 | SB | BOLD:AEE0880 |
| *Tortricidae Sp8* | 1 | 2 |  |  |  |  |  |  |  | 3 | SB | BOLD:AEE6490 |
| *Tortricidae Sp21* |  |  | 2 | 3 | 3 |  |  |  | 1 | 9 | SB | BOLD:AEE3548 |
| *Tortricidae Sp22* |  | 4 | 15 |  |  |  |  |  | 3 | 22 | SB | BOLD:AEH8919 |

**Table S5** Tree species and the a) caterpillar abundance and b) caterpillar richness across the whole vertical forest gradient (total abun./total richness) and for each vertical stratum (0-5m, 5-10m, 10-15m, 15-20m, 20-25m, 25-30m, 30-35m, 35-40, and non-assigned (NA) within the 0.1 ha plot.

**a)**

| **Species** | **Total abun.** | **Stratum** | | | | | | | | |
| --- | --- | --- | --- | --- | --- | --- | --- | --- | --- | --- |
|  |  | **0-5m** | **5-10m** | **10-15m** | **15-20m** | **20-25m** | **25-30m** | **30-35m** | **35-40m** | **NA** |
| *Antiaris africana* | 0 | 0 | 0 | 0 | 0 | 0 | 0 | 0 | 0 | 0 |
| *Celtis adolfi-friderici* | 9 | 6 | 1 | 0 | 0 | 2 | 0 | 0 | 0 | 0 |
| *Celtis mildbraedii* | 28 | 4 | 2 | 1 | 1 | 10 | 8 | 2 | 0 | 2 |
| *Celtis philippensis* | 284 | 0 | 15 | 100 | 26 | 52 | 77 | 14 | 0 | 27 |
| *Chrysophyllum boukokoensis* | 80 | 3 | 6 | 15 | 54 | 2 | 0 | 0 | 0 | 1 |
| *Chrysophyllum perpulchrum* | 73 | 8 | 2 | 11 | 46 | 6 | 0 | 0 | 0 | 5 |
| *Chytranthus sp.1* | 2 | 1 | 1 | 0 | 0 | 0 | 0 | 0 | 0 | 0 |
| *Cola gigantea* | 24 | 0 | 10 | 0 | 14 | 0 | 0 | 0 | 0 | 3 |
| *Copaifera mildbraedii* | 1 | 1 | 0 | 0 | 0 | 0 | 0 | 0 | 0 | 0 |
| *Drypetes aframensis* | 5 | 1 | 2 | 2 | 0 | 0 | 0 | 0 | 0 | 0 |
| *Drypetes aylmeri* | 71 | 3 | 0 | 22 | 39 | 7 | 0 | 0 | 0 | 3 |
| *Drypetes capillipes* | 20 | 10 | 10 | 0 | 0 | 0 | 0 | 0 | 0 | 0 |
| *Drypetes gossweileri* | 19 | 0 | 4 | 8 | 7 | 0 | 0 | 0 | 0 | 2 |
| *Drypetes molunduana* | 12 | 0 | 3 | 9 | 0 | 0 | 0 | 0 | 0 | 0 |
| *Drypetes sp. 5* | 8 | 1 | 7 | 0 | 0 | 0 | 0 | 0 | 0 | 0 |
| *Drypetes sp.2* | 7 | 0 | 6 | 1 | 0 | 0 | 0 | 0 | 0 | 0 |
| *Entandrophragma candollei* | 4 | 0 | 3 | 1 | 0 | 0 | 0 | 0 | 0 | 0 |
| *Ficus sp.1* | 11 | 0 | 0 | 1 | 6 | 3 | 1 | 0 | 0 | 1 |
| *Garcinia ovalifolia* | 3 | 0 | 0 | 3 | 0 | 0 | 0 | 0 | 0 | 0 |
| *Greenwayodendron suaveolens* | 8 | 1 | 1 | 6 | 0 | 0 | 0 | 0 | 0 | 0 |
| *Hylodendron gabunense* | 276 | 3 | 3 | 12 | 70 | 124 | 49 | 13 | 2 | 18 |
| *Mallotus oppositifolius* | 3 | 1 | 2 | 0 | 0 | 0 | 0 | 0 | 0 | 0 |
| *Mansonia altissima* | 0 | 0 | 0 | 0 | 0 | 0 | 0 | 0 | 0 | 0 |
| *Massularia acuminata* | 0 | 0 | 0 | 0 | 0 | 0 | 0 | 0 | 0 | 0 |
| *Nesogordonia papaverifera* | 5 | 0 | 1 | 0 | 4 | 0 | 0 | 0 | 0 | 1 |
| *Olax subscorpioidea* | 30 | 6 | 12 | 12 | 0 | 0 | 0 | 0 | 0 | 0 |
| *Pancovia pedicellaris* | 6 | 1 | 5 | 0 | 0 | 0 | 0 | 0 | 0 | 0 |
| *Pausinystalia macroceras* | 9 | 0 | 2 | 7 | 0 | 0 | 0 | 0 | 0 | 0 |
| *Pentaclethra macrophylla* | 6 | 0 | 0 | 6 | 0 | 0 | 0 | 0 | 0 | 0 |
| *Petersianthus macrocarpus* | 1 | 0 | 1 | 0 | 0 | 0 | 0 | 0 | 0 | 0 |
| *Pterocarpus soyauxii* | 38 | 0 | 0 | 0 | 0 | 0 | 3 | 21 | 14 | 10 |
| *Rothmannia lateriflora* | 44 | 0 | 12 | 12 | 20 | 0 | 0 | 0 | 0 | 3 |
| *Scottelia klainea* | 3 | 0 | 0 | 2 | 1 | 0 | 0 | 0 | 0 | 0 |
| *Staudtia kamerunensis* | 13 | 1 | 5 | 1 | 4 | 2 | 0 | 0 | 0 | 0 |
| *Sterculia rhinopetala* | 46 | 0 | 2 | 0 | 0 | 2 | 3 | 16 | 23 | 5 |
| *Strombosia grandifolia* | 85 | 0 | 11 | 74 | 0 | 0 | 0 | 0 | 0 | 9 |
| *Strombosia pustulata* | 8 | 0 | 2 | 2 | 4 | 0 | 0 | 0 | 0 | 0 |
| *Terminalia superba* | 77 | 0 | 2 | 0 | 0 | 10 | 28 | 25 | 12 | 16 |
| *Tricalysia pangoli* | 6 | 0 | 6 | 0 | 0 | 0 | 0 | 0 | 0 | 0 |
| *Trichilia prieuria* | 87 | 7 | 49 | 23 | 8 | 0 | 0 | 0 | 0 | 8 |
| *Trilepisium madagascariense* | 0 | 0 | 0 | 0 | 0 | 0 | 0 | 0 | 0 | 0 |
| *Uvariastrum pierreanum* | 80 | 27 | 53 | 0 | 0 | 0 | 0 | 0 | 0 | 4 |
| *Xylopia acutiflora* | 3 | 0 | 3 | 0 | 0 | 0 | 0 | 0 | 0 | 0 |

**b)**

| **Species** | **Total richness** | **Stratum** | | | | | | | | |
| --- | --- | --- | --- | --- | --- | --- | --- | --- | --- | --- |
|  |  | **0-5m** | **5-10m** | **10-15m** | **15-20m** | **20-25m** | **25-30m** | **30-35m** | **35-40m** | **NA** |
| *Antiaris africana* | 0 | 0 | 0 | 0 | 0 | 0 | 0 | 0 | 0 | 0 |
| *Celtis adolfi-friderici* | 4 | 3 | 1 | 0 | 0 | 2 | 0 | 0 | 0 | 0 |
| *Celtis mildbraedii* | 19 | 3 | 2 | 1 | 1 | 7 | 7 | 2 | 0 | 2 |
| *Celtis philippensis* | 38 | 0 | 5 | 17 | 12 | 18 | 24 | 7 | 0 | 11 |
| *Chrysophyllum boukokoensis* | 23 | 3 | 5 | 11 | 13 | 2 | 0 | 0 | 0 | 1 |
| *Chrysophyllum perpulchrum* | 19 | 2 | 1 | 2 | 18 | 3 | 0 | 0 | 0 | 5 |
| *Chytranthus sp.1* | 2 | 1 | 1 | 0 | 0 | 0 | 0 | 0 | 0 | 0 |
| *Cola gigantea* | 6 | 0 | 2 | 0 | 4 | 0 | 0 | 0 | 0 | 2 |
| *Copaifera mildbraedii* | 1 | 1 | 0 | 0 | 0 | 0 | 0 | 0 | 0 | 0 |
| *Drypetes aframensis* | 1 | 0 | 1 | 1 | 0 | 0 | 0 | 0 | 0 | 0 |
| *Drypetes aylmeri* | 16 | 3 | 0 | 2 | 15 | 5 | 0 | 0 | 0 | 3 |
| *Drypetes capillipes* | 6 | 3 | 5 | 0 | 0 | 0 | 0 | 0 | 0 | 0 |
| *Drypetes gossweileri* | 11 | 0 | 4 | 7 | 4 | 0 | 0 | 0 | 0 | 2 |
| *Drypetes molundua* | 5 | 0 | 1 | 4 | 0 | 0 | 0 | 0 | 0 | 0 |
| *Drypetes sp. 5* | 6 | 1 | 5 | 0 | 0 | 0 | 0 | 0 | 0 | 0 |
| *Drypetes sp. 2* | 5 | 0 | 5 | 1 | 0 | 0 | 0 | 0 | 0 | 0 |
| *Entandrophragma candollei* | 2 | 0 | 1 | 1 | 0 | 0 | 0 | 0 | 0 | 0 |
| *Ficus sp. 1* | 3 | 0 | 0 | 1 | 1 | 2 | 1 | 0 | 0 | 1 |
| *Garcinia ovalifolia* | 2 | 0 | 0 | 2 | 0 | 0 | 0 | 0 | 0 | 0 |
| *Greenwayodendron suaveolens* | 6 | 1 | 1 | 5 | 0 | 0 | 0 | 0 | 0 | 0 |
| *Hylodendron gabunense* | 64 | 3 | 3 | 8 | 28 | 39 | 26 | 7 | 2 | 17 |
| *Mallotus oppositifolius* | 1 | 1 | 1 | 0 | 0 | 0 | 0 | 0 | 0 | 0 |
| *Mansonia altissima* | 0 | 0 | 0 | 0 | 0 | 0 | 0 | 0 | 0 | 0 |
| *Massularia acuminata* | 0 | 0 | 0 | 0 | 0 | 0 | 0 | 0 | 0 | 0 |
| *Nesogordonia papaverifera* | 4 | 0 | 1 | 0 | 4 | 0 | 0 | 0 | 0 | 1 |
| *Olax subscorpioidea* | 17 | 6 | 11 | 7 | 0 | 0 | 0 | 0 | 0 | 0 |
| *Pancovia pedicellaris* | 4 | 1 | 4 | 0 | 0 | 0 | 0 | 0 | 0 | 0 |
| *Pausinystalia macroceras* | 3 | 0 | 1 | 3 | 0 | 0 | 0 | 0 | 0 | 0 |
| *Pentaclethra macrophylla* | 3 | 0 | 0 | 3 | 0 | 0 | 0 | 0 | 0 | 0 |
| *Petersianthus macrocarpus* | 1 | 0 | 1 | 0 | 0 | 0 | 0 | 0 | 0 | 0 |
| *Pterocarpus soyauxii* | 21 | 0 | 0 | 0 | 0 | 0 | 3 | 13 | 10 | 7 |
| *Rothmannia lateriflora* | 22 | 0 | 5 | 7 | 14 | 0 | 0 | 0 | 0 | 2 |
| *Scottelia klainea* | 3 | 0 | 0 | 2 | 1 | 0 | 0 | 0 | 0 | 0 |
| *Staudtia kamerunensis* | 9 | 1 | 3 | 1 | 4 | 2 | 0 | 0 | 0 | 0 |
| *Sterculia rhinopetala* | 21 | 0 | 2 | 0 | 0 | 2 | 3 | 9 | 9 | 4 |
| *Strombosia grandifolia* | 12 | 0 | 3 | 12 | 0 | 0 | 0 | 0 | 0 | 4 |
| *Strombosia pustulata* | 4 | 0 | 1 | 2 | 3 | 0 | 0 | 0 | 0 | 0 |
| *Terminalia superba* | 18 | 0 | 2 | 0 | 0 | 6 | 9 | 12 | 6 | 5 |
| *Tricalysia pangoli* | 3 | 0 | 3 | 0 | 0 | 0 | 0 | 0 | 0 | 0 |
| *Trichilia prieuria* | 35 | 4 | 23 | 15 | 7 | 0 | 0 | 0 | 0 | 6 |
| *Uvariastrum pierreanum* | 15 | 9 | 12 | 0 | 0 | 0 | 0 | 0 | 0 | 4 |
| *Trilepisium madagascariense* | 0 | 0 | 0 | 0 | 0 | 0 | 0 | 0 | 0 | 0 |
| *Xylopia acutiflora* | 3 | 0 | 3 | 0 | 0 | 0 | 0 | 0 | 0 | 0 |

**Table S6** Total number of individuals per tree species (abundance), total surface area (trunk + leaf surface area [m^2^]), total number of caterpillar individuals (abundance) and caterpillar species (species richness) for each tree species across the entire vertical forest gradient.

| **Trees within 0.1 ha plot** | | | | **Caterpillars** | |
| --- | --- | --- | --- | --- | --- |
| **Family** | **Species** | **Abundance** | **Surface area (m^2^)** | **Abundance** | **Species richness** |
| *Moraceae* | *Antiaris africana* | 1 | 37.38 | 0 | 0 |
| *Cannabaceae* | *Celtis adolfi-friderici* | 2 | 48.35 | 9 | 4 |
| *Cannabaceae* | *Celtis mildbraedii* | 4 | 175.54 | 28 | 19 |
| *Cannabaceae* | *Celtis philippensis* | 5 | 506.04 | 284 | 38 |
| *Sapotaceae* | *Chrysophyllum boukokoensis* | 5 | 261.97 | 80 | 23 |
| *Sapotaceae* | *Chrysophyllum perpulchrum* | 4 | 128.00 | 73 | 19 |
| *Sapindaceae* | *Chytranthus sp.1* | 2 | 32.08 | 2 | 2 |
| *Malvaceae* | *Cola gigantea* | 4 | 46.78 | 24 | 6 |
| *Fabaceae* | *Copaifera mildbraedii* | 2 | 5.81 | 1 | 1 |
| *Putranjivaceae* | *Drypetes aframensis* | 1 | 108.06 | 5 | 2 |
| *Putranjivaceae* | *Drypetes aylmeri* | 2 | 87.66 | 71 | 16 |
| *Putranjivaceae* | *Drypetes capillipes* | 5 | 123.85 | 20 | 6 |
| *Putranjivaceae* | *Drypetes gossweileri* | 2 | 100.58 | 19 | 11 |
| *Putranjivaceae* | *Drypetes molunduana* | 1 | 18.36 | 12 | 5 |
| *Putranjivaceae* | *Drypetes sp. 2* | 1 | 19.36 | 8 | 6 |
| *Putranjivaceae* | *Drypetes sp. 5* | 5 | 66.83 | 7 | 5 |
| *Meliaceae* | *Entandrophragma candollei* | 1 | 5.82 | 4 | 2 |
| *Moraceae* | *Ficus sp. 1* | 1 | 184.80 | 11 | 3 |
| *Clusiaceae* | *Garcinia ovalifolia* | 1 | 63.93 | 3 | 2 |
| *Annonaceae* | *Greenwayodendron suaveolens* | 2 | 35.14 | 8 | 6 |
| *Fabaceae* | *Hylodendron gabunense* | 9 | 691.81 | 276 | 64 |
| *Euphorbiaceae* | *Mallotus oppositifolius* | 1 | 1.98 | 3 | 1 |
| *Malvaceae* | *Mansonia altissima* | 1 | 183.47 | 0 | 0 |
| *Rubiaceae* | *Massularia acuminata* | 1 | 4.78 | 0 | 0 |
| *Malvaceae* | *Nesogordonia papaverifera* | 2 | 37.83 | 5 | 4 |
| *Olacaceae* | *Olax subscorpioidea* | 10 | 314.21 | 30 | 17 |
| *Sapindaceae* | *Pancovia pedicellaris* | 6 | 58.70 | 6 | 4 |
| *Rubiaceae* | *Pausinystalia macroceras* | 1 | 26.62 | 9 | 3 |
| *Fabaceae* | *Pentaclethra macrophylla* | 1 | 18.46 | 6 | 3 |
| *Lecythidaceae* | *Petersianthus macrocarpus* | 1 | 3.83 | 1 | 1 |
| *Fabaceae* | *Pterocarpus soyauxii* | 1 | 343.01 | 38 | 21 |
| *Rubiaceae* | *Rothmannia lateriflora* | 5 | 108.42 | 44 | 22 |
| *Achariaceae* | *Scottelia klaineana* | 3 | 78.25 | 3 | 3 |
| *Myristicaceae* | *Staudtia kamerunensis* | 7 | 277.53 | 13 | 9 |
| *Malvaceae* | *Sterculia rhinopetala* | 1 | 206.98 | 46 | 21 |
| *Olacaceae* | *Strombosia grandifolia* | 1 | 65.69 | 85 | 12 |
| *Olacaceae* | *Strombosia pustulata* | 2 | 21.46 | 8 | 4 |
| *Combretaceae* | *Terminalia superba* | 1 | 303.89 | 77 | 18 |
| *Rubiaceae* | *Tricalysia pangolina* | 2 | 28.09 | 6 | 3 |
| *Meliaceae* | *Trichilia prieuriana* | 10 | 442.90 | 87 | 35 |
| *Moraceae* | *Trilepisium madagascariense* | 1 | 8.44 | 0 | 0 |
| *Annonaceae* | *Uvariastrum pierreanum* | 11 | 112.72 | 80 | 15 |
| *Annonaceae* | *Xylopia acutiflora* | 1 | 12.36 | 3 | 3 |
